# CD200 in dentate gyrus improves depressive-like behaviors of mice through enhancing hippocampal neurogenesis via alleviation of microglia hyperactivation

**DOI:** 10.1186/s12974-023-02836-4

**Published:** 2023-06-30

**Authors:** Xi Chen, Qian-Qian Cui, Xiao-Hai Hu, Jian Ye, Zi-Cun Liu, Yuan-Xi Mei, Fang Wang, Zhuang-Li Hu, Jian-Guo Chen

**Affiliations:** 1 Department of Pharmacology, School of Basic Medicine, Tongji Medical College, Huazhong University of Science and Technology, Wuhan, China; 2 The Key Laboratory for Drug Target Researches and Pharmacodynamic Evaluation of Hubei Province, Wuhan, China; 3 The Research Center for Depression, Tongji Medical College, Huazhong University of Science, Wuhan, 430030 China; 4 Key Laboratory of Neurological Diseases (HUST), Ministry of Education of China, Wuhan, China

**Keywords:** Depression, CD200, Chronic social defeat stress, Microglia, Neurogenesis

## Abstract

**Background:**

Neuroinflammation and microglia play critical roles in the development of depression. Cluster of differentiation 200 (CD200) is an anti-inflammatory glycoprotein that is mainly expressed in neurons, and its receptor CD200R1 is primarily in microglia. Although the CD200–CD200R1 pathway is necessary for microglial activation, its role in the pathophysiology of depression remains unknown.

**Methods:**

The chronic social defeat stress (CSDS) with behavioral tests were performed to investigate the effect of CD200 on the depressive-like behaviors. Viral vectors were used to overexpress or knockdown of CD200. The levels of CD200 and inflammatory cytokines were tested with molecular biological techniques. The status of microglia, the expression of BDNF and neurogenesis were detected with immunofluorescence imaging.

**Results:**

We found that the expression of CD200 was decreased in the dentate gyrus (DG) region of mice experienced CSDS. Overexpression of CD200 alleviated the depressive-like behaviors of stressed mice and inhibition of CD200 facilitated the susceptibility to stress. When CD200R1 receptors on microglia were knocked down, CD200 was unable to exert its role in alleviating depressive-like behavior. Microglia in the DG brain region were morphologically activated after exposure to CSDS. In contrast, exogenous administration of CD200 inhibited microglia hyperactivation, alleviated neuroinflammatory response in hippocampus, and increased the expression of BDNF, which in turn ameliorated adult hippocampal neurogenesis impairment in the DG induced by CSDS.

**Conclusions:**

Taken together, these results suggest that CD200-mediated alleviation of microglia hyperactivation contributes to the antidepressant effect of neurogenesis in dentate gyrus in mice.

**Supplementary Information:**

The online version contains supplementary material available at 10.1186/s12974-023-02836-4.

## Introduction

Major depressive disorder (MDD) is one of the most common psychiatric disorders [1]. Its core symptoms include despair, anhedonia, and social avoidance [2]. According to a report issued by the World Health Organization, over 350 million people around the world have depression [3]. At its worst, depression can lead to suicide [4], and the risk of suicide mortality in patients with MDD are almost 20-fold more than the general population [5]. Although many kinds of drugs have been put into clinical use for MDD patients, they are effective in only about two-thirds of people [6]. One of the bottlenecks is less of enough druggable targets. Thus, identifying more drug targets by exploring detailed mechanisms of depression becomes highly necessary.

There is growing evidence that neuroinflammation is a potentially important contributor to the pathophysiology of depression [7]. Patients with chronic inflammation often have depressive symptoms [8], and high levels of inflammatory markers, such as interleukin-6 (IL-6), have been found in MDD patients [9, 10]. Microglia are the innate immune cells that settle in the brain and are critical to the development and progression of neuroinflammation [11], they are activated in response to tissue repairment and host defense against infectious stimuli [12]. However, the activation state of microglia is complex [13]. It is unclear whether microglia activation promotes or reduces inflammation, or whether it does both, depending on the specific cytokines acting on it. When the brain is exposed to pathological stress, the activated microglia appears to secrete pro-inflammatory cytokines, chemokines and reactive oxidants, which increase the stress susceptibility and lead to depression [14, 15]. Whereas other cytokines secreted by microglia activation, for example, IL-4 and IL-13 might induce a series of downstream processes that result in effective anti-inflammatory functions [16]. IL-4 can also drive microglia to adopt a phenotype of alternative activation with high expression of Arg-1, which is essential for hippocampal neurogenesis and the resilience to stress-induced depression [17]. Therefore, finding new ways to improve neuroinflammation is crucial for the treatment of depression.

Inflammatory responses are controlled by cell–cell interaction between neurons and microglia in the central nervous system (CNS), including cluster of differentiation 200 (CD200) signaling [18–20]. CD200 is a member of the Ig superfamily of glycoproteins that expressed on neurons, endothelial cells, dendritic cells and lymphocytes [21]. It interacts with a receptor named as CD200 receptor 1 (CD200R1) that is primarily present on myeloid cells, some lymphocytes and microglial cells [22, 23]. In the neuronal homeostasis, the interaction of CD200 expressed on neurons, with the CD200R1 expressed on microglia is essential for inhibition of microglial activation and neuroinflammation [24]. The available evidence has shown that the interaction of CD200 with CD200R1 leads to the inhibition of pro-inflammatory pathways [25], maintaining microglia in an inactive, resting state [24]. Following stress, these interactions between neurons and microglia are disrupted, enabling a pro-inflammatory response [26]. However, the role of CD200/CD200R1 in depression is not currently known.

Recently, activation of microglia in depression has been revealed to have the capacity to regulate neurogenesis and play a role in the development of depression. The hyperactivated microglia secretes inflammatory mediators to impair neurogenesis, promoting the development of depression [15]. Meanwhile, the inhibition of neuroinflammation increases adult neurogenesis and ameliorates depressive-like symptoms [17, 27]. As a regulator of microglia homeostasis, CD200 has also been reported to be involved in neurogenesis. In a mouse model of Alzheimer's disease, injection of exogenous CD200 in the DG region could enhance microglia-mediated neural differentiation of neural stem cells [28]. Meanwhile, treadmill exercise could promote neurogenesis and functional recovery after stroke via activating the CD200/CD200R1 signaling pathway [29]. Nevertheless, the association of CD200, microglia and neurogenesis in depression is unclear.

Taken together, the development of depression is closely related to the activation of microglia and corresponding changes, and CD200/CD200R1 signaling plays an important role in maintaining microglia homeostasis. Therefore, the effect and mechanisms of CD200 on depression were investigated. With chronic stress, behavioral tests, molecular biology, and gene-manipulation approaches, our present study indicated that CD200 in the DG would alleviate depressive-like behaviors in mice via ameliorating the impairment of neurogenesis that was related to the activation of microglia, which may provide a potential target for the treatment of MDD.

## Materials and methods

### Animals

In this study, 7-week-old C57BL/6 J male mice were purchased from Hunan SJA Laboratory Animal (Changsha, Hunan, China). Six- to eight-month-old retired male breeder CD-1 mice were purchased from Beijing Vital River Laboratory Animal Technology (Beijing, China). All mice were housed in standard conditions (12-h light/dark cycle under stable temperature (22 ± 2 °C) and consistent humidity (50 ± 5%) with ad libitum access to food and water) and were assigned to each experimental group randomly. All procedures were approved by the animal Welfare Committee of Huazhong University of Science and Technology.

### Chronic social defeat stress (CSDS)

**Fig. 1:**
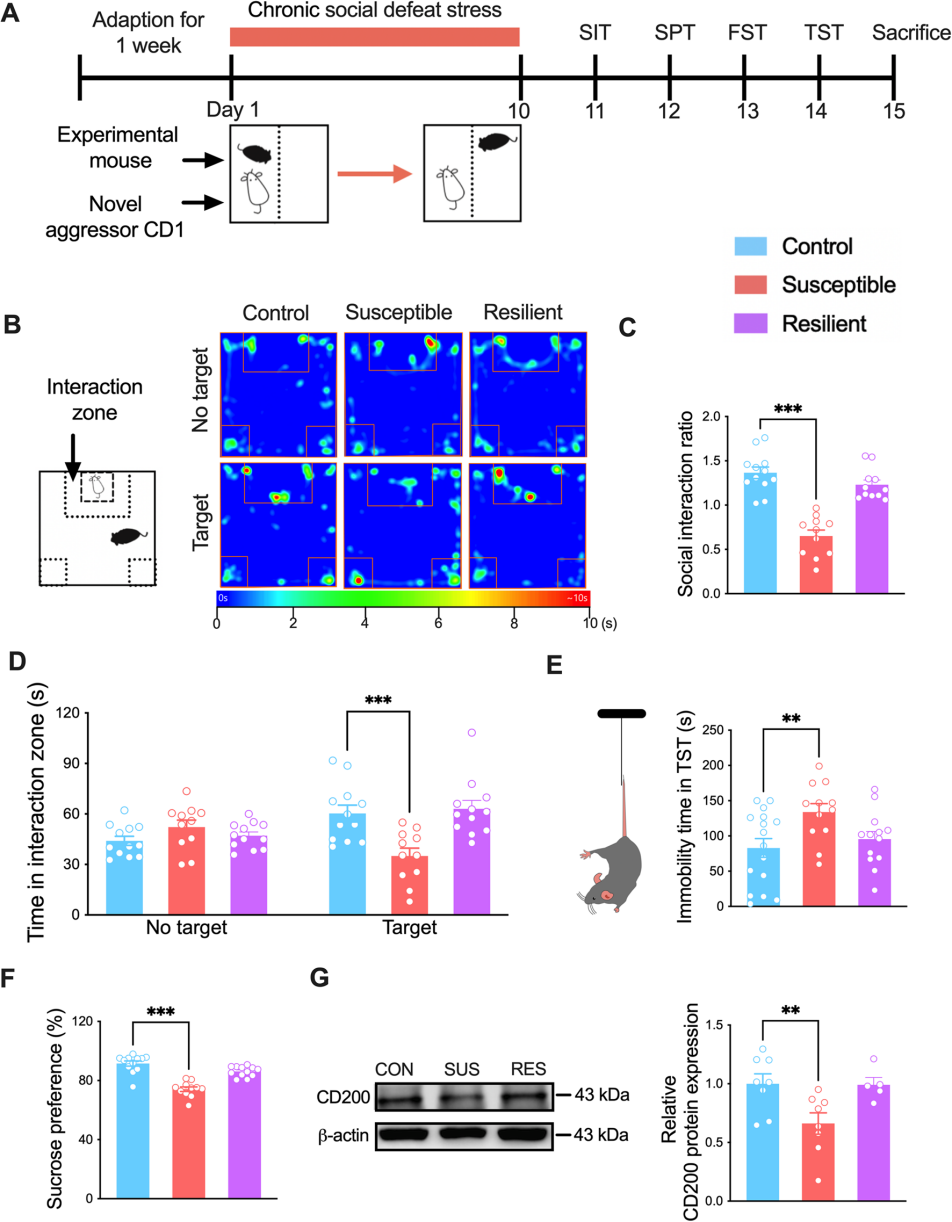
CSDS decreases the expression of CD200 protein in DG. **A** Time-line of experimental procedures and schematic representation of chronic social defeat stress. SIT, social interaction test; SPT, sucrose preference test; FST, force swimming test; TST, tail suspension test. **B** Typical heatmap of SIT in control, susceptible and resilient mice. **C** Social interaction ratio in control and stressed mice. The data showed ratio < 1 in susceptible mice and > 1 in resilient mice (*n* = 11–12 per group, one-way ANOVA with Fisher’s LSD test). **D** Social interaction time in no target and target zone in control, susceptible and resilient mice following CSDS (*n* = 11–12 per group, two-way ANOVA with Fisher’s LSD test). **E** Immobility time in TST in control, susceptible and resilient mice (*n* = 12–16 per group, one-way ANOVA with Fisher’s LSD test). **F** Sucrose preference in control, susceptible and resilient mice (*n* = 11–12 per group, one-way ANOVA with Fisher’s LSD test).** G** Protein expression of CD200 in the DG region after CSDS (*n* = 5–8 per group, one-way ANOVA with Fisher’s LSD test). Data are expressed as mean ± SEM. **P < 0.01; ***P < 0.001. CON, control; SUS, susceptible; RES, resilient. See Additional file 1: Table S2 for detailed statistical information

The CSDS protocols were performed as described previously [30, 31] and shown in Fig. 1A. C57BL/6 J mice were exposed to 5–10 min of physical aggression by an intruder male CD-1 mouse. Following the defeat session, experimental mice were housed in the same cage as the CD-1 mice on the opposite side of a transparent and perforated plexiglass divider to maintain sensory contact for 24 h. This procedure was repeated for 10 consecutive days with a new CD-1 aggressor on each day. Control mice were pair housed in similar cages, separated from one another by a perforated plexiglass divider and rotated to a new one each day for 10 d.

### Subthreshold social defeat stress (SSDS)

SSDS was designed to detect the susceptibility of mice to stress [32]. Experimental mice were exposed to a novel CD-1 aggressor for three social defeat sessions with 5 min of physical defeat followed by 15-min intervals. Immediately after the last defeat session, all experimental mice were housed to their home cages. This procedure does not induce social avoidance in normal mice and is used to reveal potential mechanisms that make the mice more sensitive to further stress [33, 34].

### Behavioral procedures

All behavioral tests were performed in adult C57BL/6 J male mice. Before the experiment began, mice were adapted to the room conditions for at least 1 h. After every test session, the apparatus was cleaned with 75% alcohol to eliminate the odor of the previous mouse.

### Social interaction test (SIT)

SIT was carried out 24 h after the last defeat session of either chronic social defeat or subthreshold social defeat. There were two trials. During the first trial, the experimental mouse was placed into a chamber (45 cm × 45 cm × 45 cm) for 2.5 min. A mesh enclosure (10 cm × 4.5 cm) was put in the interaction area, and the time that subject mouse spent in the interaction area was signed ‘no target time’. During the second trial, a novel CD-1 mouse was placed into the enclosure, and the experimental mouse was returned to the chamber for another 2.5 min [31]. This time in the interaction area was ‘target time’. ANY-maze software (Stoelting Co, Wood Dale, IL, USA) was used for video tracking. The social interaction ratio was calculated as target time/no target time, and the mice with the ratio < 1 were considered susceptible mice and the ratio > 1 were considered resilient mice.

### Sucrose preference test (SPT)

SPT was performed on days 11–12 using a two-bottle choice procedure. The mice were habituated to two bottles of water for 24 h, and the position of bottles was interchanged every 12 h to avoid biases from side preference. For testing, the bottle with 1% sucrose and water solution was renewed, and the original weight was recorded as the baseline value. After 12 h, the bottle weight was recorded as the final value. The baseline value subtracted from the final value was intake solution. The sucrose preference = (sucrose intake/total intake) × 100% [35].

### Tail suspension test (TST)

TST is a test to evaluate despair or depressive-like behavior. The mouse was suspended 20 cm above the floor with adhesive tape affixed 1.0 cm from the tip of the tail [35]. The time during which mice remained immobile over a period of 6 min was recorded.

### Forced swimming test (FST)

FST was also performed to evaluate despair or depressive-like behavior. First, a clean plastic chamber (25 cm × 18 cm diameter) was filled with water (22 ± 0.5 °C), and mice were forced to swim for 6 min [1]. The duration of immobility for the last 4 min was quantified.

### Open field test (OFT)

OFT was used to assess the motor abilities of mice. Mice were placed in a plexiglass arena (45 cm × 45 cm × 45 cm) and were allowed to explore the open field freely for 10 min [36]. The total traveled distance was scored.

### Western blotting

Western blotting was performed according to the standard protocol [37]. Brain tissues were homogenized in RIPA lysis buffer (50 mM Tris–HCl, pH 7.4, 1% NP-40, 0.5% Na-deoxycholate, 0.1% sodium dodecyl sulfate (SDS), 150 mM NaCl, 2 mM EDTA and 50 mM NaF) containing phosphatase inhibitors (Sigma-Aldrich, St. Louis, MO, USA) and protease inhibitors (Roche, Basel, Switzerland). The protein supernatant was mixed with 4 × loading buffer, then inactivated at 95 °C for 10 min. Samples were separated on 10% sodium dodecyl sulfate–polyacrylamide gels (SDS–PAGE) and then transferred onto nitrocellulose membranes. The membranes were blocked with 5% bovine serum albumin (BSA) for 1 h at room temperature, and then were incubated overnight at 4 °C with different primary antibodies against β-actin (sc-47778, 1:3000 dilution, Santa Cruz Biotechnology, California, USA), CD200 (sc-14388, 1:500 dilution, Santa Cruz Biotechnology, California, USA) and CD200R (sc-53101, 1:200 dilution, Santa Cruz Biotechnology, California, USA). On the second day, the membranes were incubated with horseradish peroxidase (HRP)-conjugated secondary antibodies (1:5000 dilution, Invitrogen, Waltham, USA) for 1 h at room temperature. The immunoreactive bands were visualized and quantified with a MicroChemi system (DNR Bio-Imaging Systems, Jerusalem, Israel). All original of key western blots and information were presented in the Additional file 1: Fig. S6.

### Stereotaxic surgery

C57BL/6 J mice were anesthetized with sodium pentobarbital (40 mg/kg, i.p.) and then were placed in the stereotaxic frame. Cannulas were implanted bilaterally to the lateral ventricle (AP = − 0.3 mm, ML =  ± 0.8 mm, DV = − 2.8 mm) or DG (AP = − 1.8 mm; ML =  ± 1.8 mm; DV = − 2.4 mm). Seven days later, recombinant mouse CD200 Fc chimera protein CF (CD200Fc) (#3355-CD-050, R&D Systems, Minnesota, USA), as a substitute for endogenous CD200, was microinjected into the lateral ventricle or DG area for 7 d. CD200 Fc was dissolved in artificial cerebrospinal fluid (ACSF) (119 mM NaCl, 1.3 mM MgSO_4_, 3.5 mM KCl, 11 mM glucose, 26.2 mM NaHCO_3_, 1 mM NaH_2_PO_4_, 2.5 mM CaCl_4_, PH = 7.4) with a final concentration of 2 μg/μl [38] and infused with a volume of 2 μl per side.

### Stereotaxic virus injection

Lentiviral vectors with siRNA sequence targeting the CD200 gene (AAGCATTGTTTCTCTGGTAAT, LV-siCD200) and CD200R1 gene (TGTGACTAAGGTGGAGGCATT, LV-siCD200R1) were constructed by Genechem Co., Ltd. (Shanghai, China). The control lentiviral vector expressing GFP (LV-GFP) was designed with a scramble sequence (TTCTCCGAACGTGTCACGT). A volume of 1 μl lentiviral vector solution was microinjected bilaterally into the DG area (AP = − 1.8 mm; ML =  ± 1.8 mm; DV = − 2.4 mm) at a rate of 100 nl/min, then the injection needle was left in place for 10 min to prevent backflow.

The adeno-associated virus (AAV) containing the gene for CD200 (AAV–CMV–βGlobin–CD200–3Flag–T2A–EGFP, AAV–CD200) or EGFP alone (AAV–CMV–βGlobin–MCS–EGFP–MCS–3FlagPoly A, AAV–GFP) was purchased from Genechem Co., Ltd. (Shanghai, China). After anesthetizing, mice were placed in a stereotaxic frame. A volume of 200 nl AAV was injected bilaterally into the DG region at 100 nl/min using a syringe pump.

### Immunofluorescence staining

Mice were anesthetized and perfused with 4% paraformaldehyde in 0.1 M sodium phosphate buffer saline (PBS). The brains were removed and post-fixed in 4% paraformaldehyde overnight at 4 °C, and then equilibrated in 15% or 30% gradient sucrose. Frozen sections were cut into 30 μm with a freezing microtome (CM1900, Leica Microsystems, Germany). For 5-bromo-2-deoxyuridine (BrdU) staining, sections were first incubated in 1 M hydrochloric acid (HCl) for 10 min and then 2 M HCl for another 10 min at room temperature and neutralized with 0.1 M sodium borate buffer (pH 8.5). Then, all tissue sections were blocked in a buffer containing 0.3% Trixon-100 and 5% BSA in PBS for 1 h, and incubated overnight with primary antibody as follows: doublecortin (DCX, #4604, 1:500 dilution, Cell Signaling Technology, Boston, USA), BrdU (ab6326, 1:1000 dilution, Abcam, Cambridge, UK), Iba1 (ab5076 and ab178846,1:200 dilution, Abcam, Cambridge, UK), CD68 (MCA1957, 1:400 dilution, Bio-Rad Laboratories, California, USA), BDNF (ab108319, 1:500 dilution, Abcam, Cambridge, UK), arginase-1 (Arg-1, sc-271430, 1:100 dilution, Santa Cruz Biotechnology, California, USA), SOX2 (AF2018, 1:100 dilution, R&D Systems, Minnesota, USA), Ki67 (ab15580, 1:500 dilution, Abcam, Cambridge, UK), GFAP (#3670, 1:500 dilution, Cell Signaling Technology, Boston, USA). After washing with PBS, slices were incubated with Alexa secondary antibodies (1:1000 dilution, Invitrogen, Waltham, USA). Slices were loaded on slides and imaged with a confocal microscope (FV1000, Olympus, Tokyo, Japan). Calculation of fluorescence intensity and 3D cell reconstruction were carried out with the Imaris 9.0 software.

### Quantification of immunoreactive cells

The number of microglia was assessed by counting Iba1 positive cell after tissue preparation as above. For labeling cells at different stages of neurogenesis, BrdU (B5002, Sigma, St Louis, Missouri, USA) was dissolved in saline at 10 mg/ml and was intraperitoneally injected at a dose of 50 mg/kg for consecutive 3 days before tissue preparation. The granular and sub-granular layers of DG in every tenth section were then selected and immunolabeled. Immunoreactive cells (Iba1, SOX2, GFAP, Ki67, DCX, BrdU) were counted on an Olympus FV1000 confocal microscope with FluoView software. Both hemispheres were counted and cell counts were averaged across both hemispheres for each section. The number of immunopositive cells was represented as the averaged cell counts per hemisection of all sections within each hippocampus [39].

### Statistical analysis

Data were expressed as the mean ± SEM and analyzed using GraphPad Prism 9.0. Statistical analysis was performed using Student’s *t* test between two-group analysis, and one-way or two-way ANOVA test followed by Fisher’s LSD test for multi-group analysis. Differences were considered statistically significant when *P* < 0.05.

## Results

### The expression of CD200 is decreased in the DG of mice exposed to CSDS

To identify the role of CD200 in the pathophysiology of MDD, CSDS was used to mimic psychopathological dimensions of depression [40] (Fig. 1A). After 10 days of stress, the susceptible mice but not the resilient mice showed social avoidance (Fig. 1B) with social interaction ratio < 1 (*F*_(2, 31)_ = 36.49, *P* < 0.001; Fig. 1C) and decreased time in the interaction zone in the SIT (*F*_(2, 64)_ = 10.83, *P* < 0.001; Fig. 1D). In addition, the susceptible mice showed increased immobility time in the TST (*F*_(2,38)_ = 4.377, *P* = 0.019; Fig. 1E) and decreased sucrose preference ratio in SPT (*F*_(2, 32)_ = 35.51, *P* < 0.001; Fig. 1F). Next, we examined the changes in CD200 mRNA levels in the medial prefrontal cortex (mPFC) and hippocampus, both of which were closely associated with stress and depression. There was no obvious difference in mPFC between control and susceptible mice (Additional file 1: Fig. S1A, *P* = 0.280); however, the level of CD200 in the hippocampus of susceptible mice was decreased (Additional file 1: Fig. S1B, *P* = 0.021). Thus, the hippocampal subregion was further examined, and an obvious decline of CD200 mRNA was only observed in the DG subregion of susceptible mice (Additional file 1: Fig. S1C, *P* < 0.001; Fig. S1D, *P* = 0.091; Fig. S1E, *P* = 0.828). Meanwhile, CD200 protein in DG was also reduced in susceptible mice (*F*_(2, 18)_ = 5.339, *P* = 0.015; Fig. 1G). Together, these results demonstrate that chronic social defeat stress induces a reduction of CD200 expression in the DG of susceptible mice.

## Upregulation of CD200 in the DG alleviates the depressive-like behaviors of mice induced by CSDS

We then investigated the effect of CD200 on chronic stress-induced depressive-like behaviors. Following CSDS in C57BL/6 J male mice, CD200Fc (2 μg/μl) was infused with a volume of 2 μl per side daily into the lateral ventricle for consecutive 7 d (Additional file 1: Fig. S2A). The SIT results showed that intracerebroventricular injection of CD200Fc ameliorated the decreased social interaction ratio induced by CSDS (Additional file 1: CD200 Factor: *F*_(1, 32)_ = 12.66, *P* = 0.001; Fig. S2B). Moreover, despaired behaviors evaluated by TST and FST were also tested, and the results showed that the increased immobility time of stressed mice in TST and FST was reduced by CD200Fc infusion (Additional file 1: Fig. S2C *F*_(1, 35)_ = 9.247, *P* = 0.004; Fig. S2D Stress Factor *F*_(1, 39)_ = 13.45, *P* < 0.001). To exclude the influence of locomotor activity, OFT was examined and the total distance was calculated, it was found that CD200Fc supplementation had no effect on locomotor ability (Additional file 1: Fig. S2E* F*_(1, 41)_ = 0.1198, *P* = 0.731). These results indicate that the exogenous injection of CD200Fc to lateral ventricle improves depressive-like behaviors of stressed mice.

**Fig. 2:**
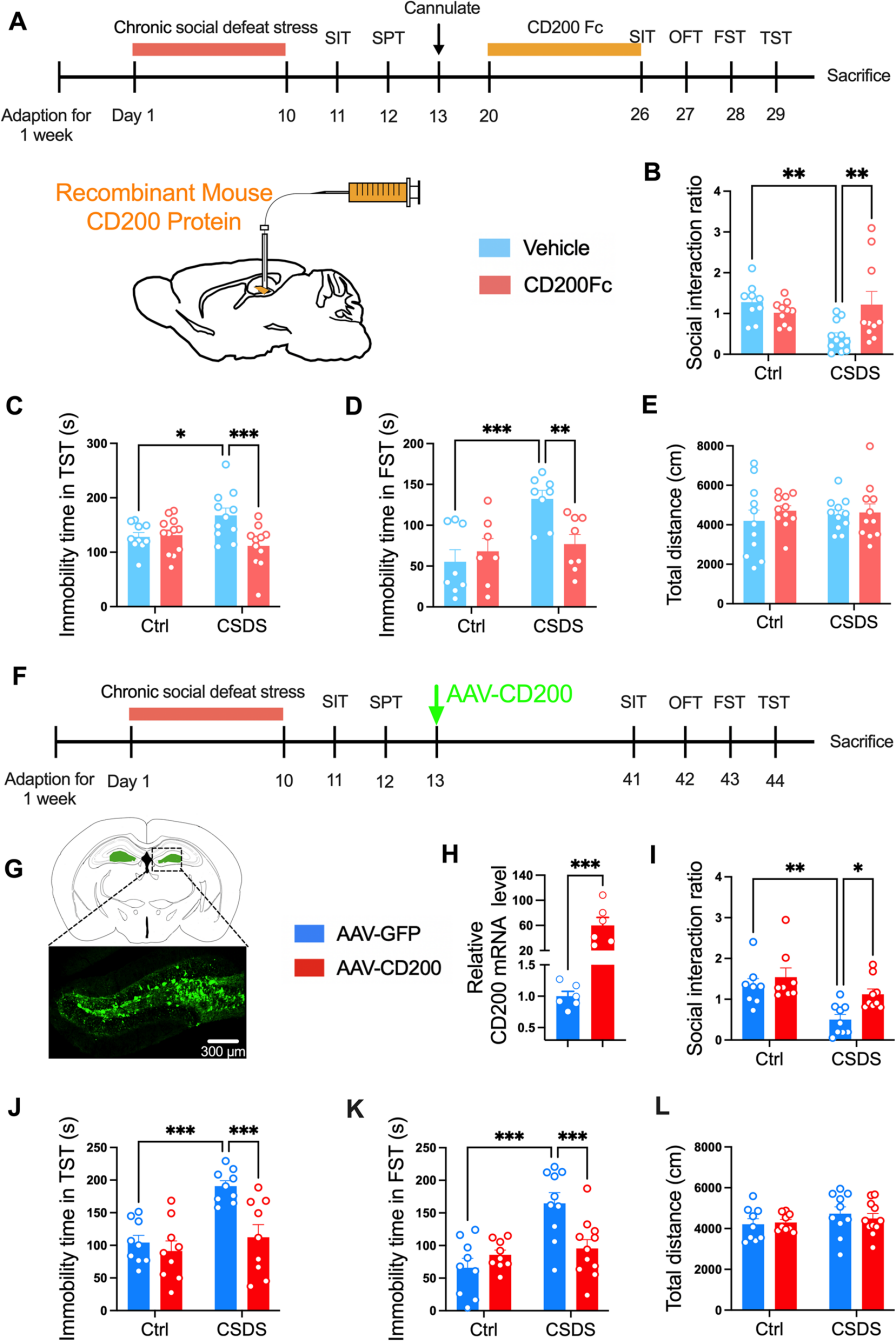
Upregulation of CD200 in the DG alleviates the depressive-like behaviors of mice induced by CSDS. **A** Experimental timelines for CSDS procedure, CD200Fc administration and behavioral testing. **B** Social interaction ratio in the SIT of control and CSDS mice with exogenous CD200Fc injection (*n* = 9–12 per group, two-way ANOVA with Fisher’s LSD test). **C**, **D** Immobility time in the TST (**C**) and FST (**D**) of control and CSDS mice with exogenous CD200Fc injection (TST, *n* = 10–12 per group; FST, *n* = 7–8 per group, two-way ANOVA with Fisher’s LSD test). **E** Total distance in the OFT of control and CSDS mice with exogenous CD200Fc injection (*n* = 11 for each group, two-way ANOVA with Fisher’s LSD test). **F** Experimental timelines for CSDS procedure, AAV–CD200 stereotaxical injection and behavioral testing. **G** Representative image showing the expression of AAV–CD200 in DG 4 weeks after virus vector injection. Scale bar: 300 μm. **H** Analysis of CD200 mRNA levels in DG after injection with AAV–CD200 virus. (*n* = 6 per group, Student’s *t* test). **I** Social interaction ratio in the SIT of control and CSDS mice with AAV–CD200 infusion (*n* = 8–9 per group, two-way ANOVA with Fisher’s LSD test). **J**, **K** Immobility time in the TST (J) and FST (K) of control and CSDS mice with AAV-CD200 infusion (TST, *n* = 9 for each group; FST, *n* = 9–11 per group, two-way ANOVA with Fisher’s LSD test). **L** Total distance in the OFT of control and CSDS mice with AAV-CD200 infusion (*n* = 9–11 per group, two-way ANOVA with Fisher’s LSD test). Data are expressed as mean ± SEM. *P < 0.05; **P < 0.01; ***P < 0.001. Ctrl, control. See Additional file 1: Table S2 for detailed statistical information

We further infused CD200Fc into the DG region specially for consecutive 7 d after CSDS and examined depressive-like behavioral changes of mice (Fig. 2A). CD200Fc delivery into the DG increased the social interaction ratio in the SIT (*F*_(1, 37)_ = 7.891, *P* = 0.008; Fig. 2B), and decreased immobility time in the TST and FST in susceptible mice (*F*_(1, 40)_ = 7.197, *P* = 0.011, Fig. 2C; *F*_(1, 27)_ = 6.625, *P* = 0.016, Fig. 2D), without affecting locomotor activity in the OFT (*F*_(1, 40)_ = 0.2664, *P* = 0.609; Fig. 2E). These results suggest that exogenous injection of CD200Fc to the DG region ameliorates chronic stress-induced depressive-like behaviors in mice.

Then, the AAV–CD200 with green fluorescence protein (GFP) was constructed to evaluate the effect of CD200 in the DG on depressive-like behaviors of mice (Fig. 2F). The accuracy of the stereotaxic injection site was confirmed by the green fluorescence of the virally expressed GFP protein (Fig. 2G) and the overexpression efficiency was verified by PCR detection (*P* < 0.001, Fig. 2H). It was shown that AAV–CD200 alleviated the social avoidance in susceptible mice (CD200 Factor: *F*_(1, 30)_ = 14.14, *P* < 0.001; Fig. 2I). Furthermore, mice injected with AAV–CD200 displayed reduced immobility time in the TST and FST when compared with the AAV–GFP group after CSDS (*F*_(1, 32)_ = 5.388, *P* = 0.027, Fig. 2J; *F*_(1, 35)_ = 10.54, *P* = 0.003, Fig. 2K). The total distance traveled was not affected in the AAV–CD200 group (*F*_(1, 35)_ = 0.3828, *P* = 0.540; Fig. 2L), indicating that the reduction of immobility was not likely due to motor defects. These results demonstrate that overexpression of CD200 in the DG improves depressive-like behaviors of mice induced by CSDS.

### Knockdown of CD200 in the DG region facilitates stress susceptibility of mice

**Fig. 3:**
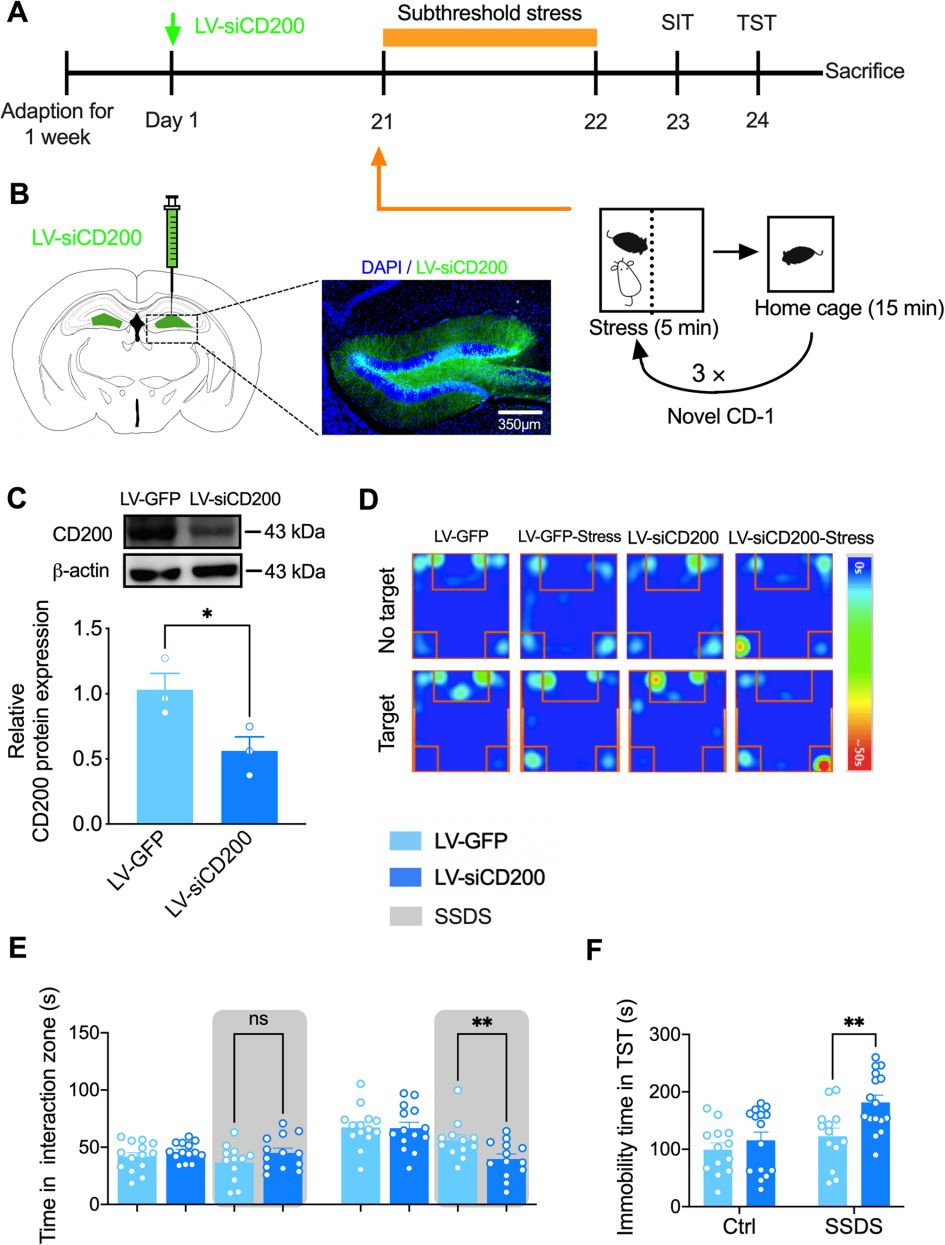
Knockdown of CD200 in DG region facilitates stress susceptibility of mice. **A** Experimental timeline for LV-siCD200 stereotaxical injection, SSDS procedure, and behavioral testing. **B** Representative image showing the expression of LV-siCD200 in DG 3 weeks after virus vector injection. Scale bar: 350 μm. **C** Immunoblot analysis of CD200 protein expression in the DG region of mice injected with LV-siCD200 (*n* = 3 per group, Student’s *t* test).** D** Heatmap of SIT in mice injected with LV-siCD200 or LV-GFP to DG, together with or without exposure to SSDS.** E** Social interaction time in the SIT of mice injected with LV-siCD200 and exposed to SSDS (*n* = 12–14 per group, one-way ANOVA with Fisher’s LSD test). **F** Immobility time in the TST of mice injected with LV-siCD200 and exposed to SSDS (*n* = 13–16 per group, two-way ANOVA with Fisher’s LSD test). Data are expressed as mean ± SEM. ns: not statistically significant; *P < 0.05; **P < 0.01. Ctrl, control. See Additional file 1: Table S2 for detailed statistical information

To examine the stress susceptibility of mice after downregulation of CD200 expression in DG, the LV-siCD200 was constructed and injected into the DG region, 3 weeks later, SSDS was performed and behaviors were detected on mice (Fig. 3A). The accuracy of the injection site was confirmed by immunofluorescence staining of GFP (Fig. 3B). Injection of LV-siCD200 in the DG region decreased the expression of CD200 protein by approximately 47% (*P* = 0.046, Fig. 3C). Furthermore, the result of SIT revealed that LV-siCD200-treated mice exhibited decreased interaction time with a social target after SSDS, which represented a pro-depressive-like effect compared with LV-GFP-treated mice (*F*_(7, 98)_ = 8.126, *P* < 0.001; Fig. 3D, E). The result of TST also showed that the immobility time in the LV-siCD200 mice exposed to SSDS was increased compared with LV-GFP group (LV-siCD200 Factor: *F*_(1, 53)_ = 11.01, *P* = 0.002; Fig. 3F). These results suggest that the reduction of CD200 protein in DG facilitates the susceptibility of mice to stress.

### CD200 alleviates CSDS-induced depressive-like behavior via binding to microglial CD200R1

**Fig. 4:**
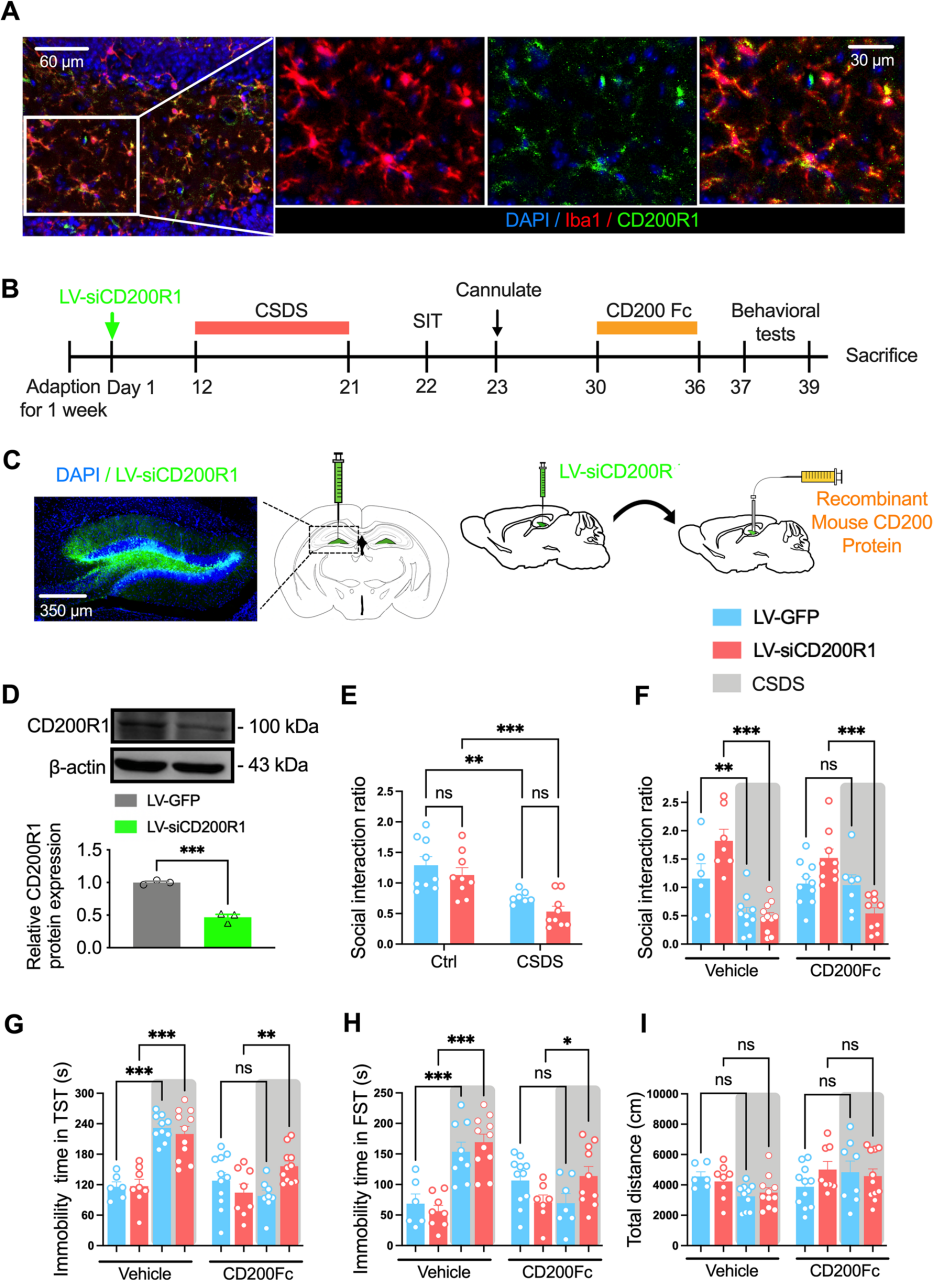
CD200 alleviates CSDS-induced depressive-like behaviors through binding to CD200R1 on microglia. **A** Representative immunostaining for CD200R1 (green)/Iba1 (red)/DAPI (blue) in the DG. Scale bars: 60 μm (right), 30 μm (left).** B** Experimental timeline for LV-siCD200R1 stereotaxically injection, CSDS procedure, CD200Fc administration and behavioral testing. **C** Representative image showing the expression of LV-siCD200R1 expression in DG 3 weeks after virus vector injection. Scale bar: 350 μm. **D** Immunoblot analysis of CD200R1 protein expression in the DG region of mice injected with LV-siCD200R1 (*n* = 3 per group, Student’s *t* test). **E** Social interaction ratio in the SIT of control and CSDS mice injected with LV-siCD200R1 or LV-GFP (*n* = 7–9 per group, two-way ANOVA with Fisher’s LSD test). **F** Analysis of social interaction ratio in the SIT of control and CSDS mice injected with LV-siCD200R1 and CD200Fc (The CD200Fc administration group: *n* = 7–10 per group; The vehicle administration group: *n* = 6–11 per group, one-way ANOVA with Fisher’s LSD test). **G** Analysis of immobility time in TST of control and CSDS mice injected with LV-siCD200R1 and CD200Fc (The CD200Fc administration group: *n* = 8–11 per group; The vehicle administration group: *n* = 6–11 per group, one-way ANOVA with Fisher’s LSD test). **H** Analysis of immobility time in FST of control and CSDS mice injected with LV-siCD200R1 and CD200Fc (The CD200Fc administration group: *n* = 7–11 per group; The vehicle administration group; *n* = 6–11 per group, one-way ANOVA with Fisher’s LSD test). **I** Analysis of total distance in OFT of control and CSDS mice after injected with LV-siCD200R1 and CD200Fc (The CD200Fc administration group: *n* = 8–11 per group; The vehicle administration group; *n* = 6–11 per group, one-way ANOVA with Fisher’s LSD test). Data are expressed as mean ± SEM. ns: not statistically significant; *P < 0.05; **P < 0.01; ***P < 0.001. Ctrl, control. See Additional file 1: Table S2 for detailed statistical information

CD200R1 is the major receptor of CD200, which is mainly distributed on the microglia [41]. Here, the immunofluorescence staining with Iba1 and CD200R1 was performed and it was shown that CD200R1 was expressed in microglia (Fig. 4A). We then asked whether CD200R1 participated in the antidepressant effects of CD200. The LV-siCD200R1 with GFP was used here to knockdown CD200R1 in the DG region, and then CD200Fc was injected into DG to compare the depressive-like behaviors (Fig. 4B). The green fluorescence confirmed the accuracy of the injection site (Fig. 4C), and after 3 weeks of LV-siCD200R1 injection, the protein level of CD200R1 in DG was decreased by approximately 54% (*P* < 0.001, Fig. 4D). Then, the behavioral tests were performed. In the SIT, there were no significant differences in the social interaction ratio between LV-GFP and LV-siCD200R1 group, both of which were decreased after CSDS (LV-siCD200R1 Factor: *F*_(1, 30)_ = 25.73, *P* < 0.001; Fig. 4E). However, CD200Fc infusion only rescued the social avoidance of stressed mice in the LV-GFP group, the social interaction ratio was still reduced in stressed mice of the LV-siCD200R1 group after CD200Fc infusion (*F*_(7, 61)_ = 11.42, *P* < 0.001; Fig. 4F). Meanwhile, the increased immobility time in the TST and FST of LV-siCD200R1 mice after CSDS could not be improved with CD200Fc administration (*F*_(7, 65)_ = 15.97, *P* < 0.001, Fig. 4G; *F*_(7, 61)_ = 9.369, *P* < 0.001; Fig. 4H). Moreover, the locomotor activity of mice was kept unchanged in all groups (*F*_(7, 65)_ = 2.061, *P* = 0.061; Fig. 4I). Administration of LV-siCD200R1 with vehicle did not affect the behavioral profile of mice (Fig. 4E–I). Therefore, these results suggest that selective knockdown of CD200R1 in DG blocks the antidepressant-like behaviors of CD200Fc, indicating that the effects of CD200 are mediated by activation of CD200R1.

### CD200 ameliorates CSDS-induced microglia hyperactivation and hippocampal neuroinflammation

**Fig. 5:**
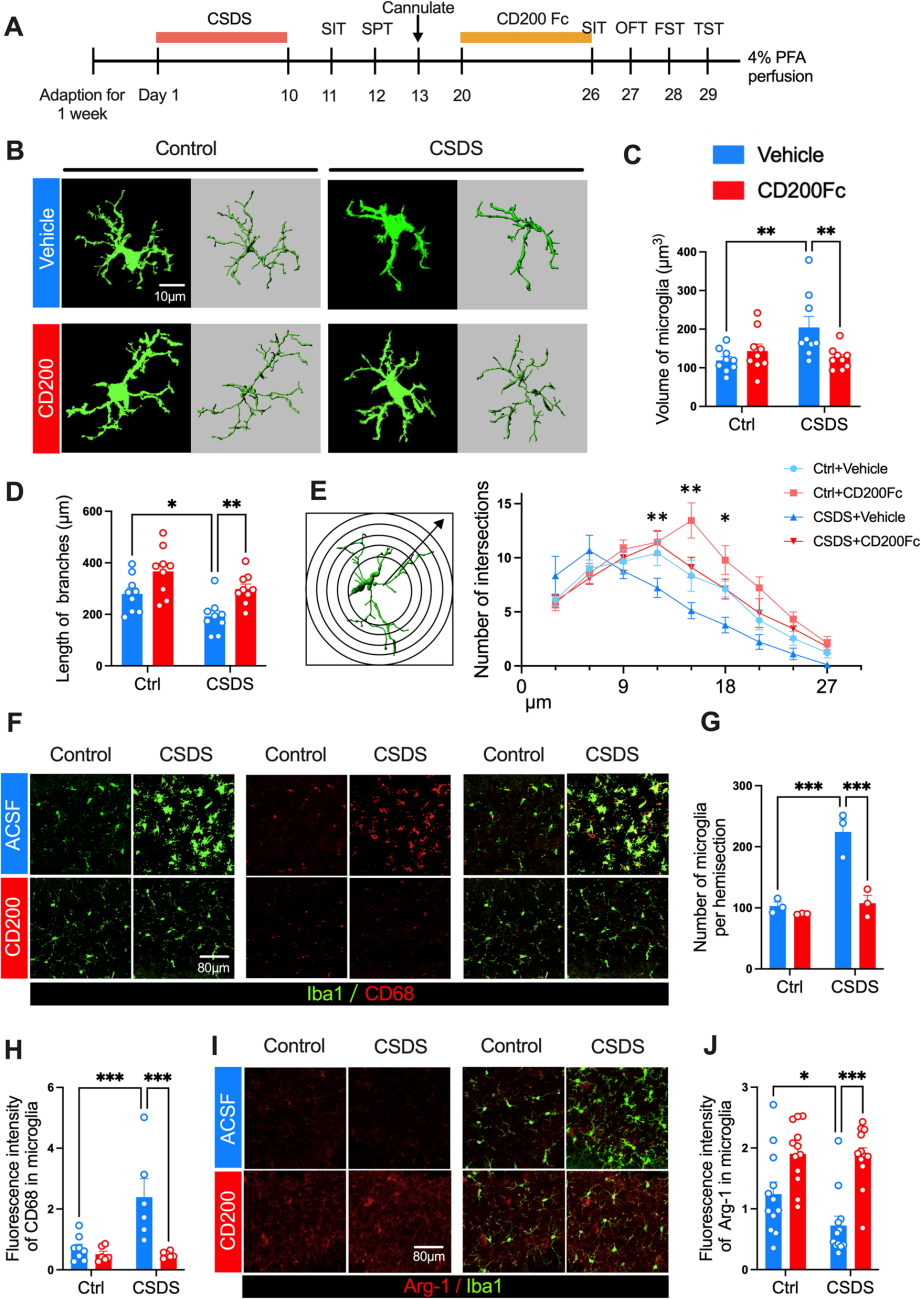
CD200 ameliorates CSDS-induced microglia hyperactivation and hippocampal neuroinflammation. **A** Experimental timelines for CSDS procedure, CD200Fc administration and behavioral testing. **B** Representative 3D reconstructions showing the microglial morphology in DG of control and CSDS mice with Iba1 staining (green). Scale bars: 10 μm. **C–E** Volume of microglia (**C**), length of branches (**D**), the number of intersections (**E**) in the control and CSDS group after CD200Fc administration (*n* = 9 sections/group from 3 mice, one-way ANOVA with Fisher’s LSD test).** F** Representative immunostaining for Iba1 (green)/CD68 (red) in DG of control and CSDS mice. Scale bars: 80 μm. **G** Number of microglia in the control and CSDS group after CD200Fc administration (*n* = 3 mice per group, two-way ANOVA with Fisher’s LSD test).** H** Fluorescence intensity of CD68 in microglia in the control and CSDS group after CD200Fc administration (*n* = 6–8 sections from 3 mice per group, two-way ANOVA with Fisher’s LSD test). **I** Representative immunostaining for Iba1 (green)/Arg-1 (red) in DG of control and CSDS mice. Scale bars: 80 μm. **J** Fluorescence intensity of Arg-1 in microglia in the control and CSDS group after CD200Fc administration (*n* = 12 sections from 3 mice per group, two-way ANOVA with Fisher’s LSD test). Data are expressed as mean ± SEM. *P < 0.05; **P < 0.01. ***P < 0.001. Ctrl, control. See Additional file 1: Table S2 for detailed statistical information

Since disruption of the CD200–CD200R interaction activates microglia [25], the effect of CD200 on microglial status was investigated. The experimental procedure is shown in Fig. 5A. The 3D reconstructions of the microglia in the DG region were applied to reveal distinct cellular morphological changes (Fig. 5B). After exposure to CSDS, microglial arborization altered in susceptible mice, manifesting as enlargement of microglia volume and reduction in length of cell branches, as well as decrease in the number of intersections in the DG, and all above morphological changes in microglia in the susceptible group could be relieved by exogenous CD200Fc infusion (*F*_(1, 32)_ = 8.187, *P* = 0.007, Fig. 5C; CD200Fc Factor: *F*_(1, 32)_ = 10.21, *P* = 0.003, Fig. 5D; *F*_(24, 288)_ = 2.548, *P* < 0.001, Fig. 5E).

To further evaluate the microglia state, immunofluorescence staining was performed to label activated microglia with CD68 and a non-inflammatory phenotype of microglia with Arg-1 [42] to assess the microglial activation status [17, 43]. First, the number of microglia, marked with Iba1 in the DG region was increased after CSDS, which was reduced by CD200Fc supplementation (*F*_(1, 8)_ = 16.19, *P* = 0.004; Fig. 5F, G). Correspondingly, the fluorescence intensity of CD68 was enhanced, while the Arg-1 fluorescence intensity within microglia was decreased after CSDS, and exogenous CD200Fc administration restored the fluorescence intensity of CD68 and Arg-1 (*F*_(1, 22)_ = 8.119, *P* = 0.009, Fig. 5H; CD200Fc Factor: *F*_(1, 44)_ = 3.014, *P* = 0.090, Fig. 5I, J). Then, the gene expressions of a variety of pro-inflammatory and anti-inflammatory cytokines were detected after CSDS to determine the effect of AAV–CD200 on the inflammatory response of hippocampus (Additional file 1: Fig. S3A). After exposure to CSDS, injection of AAV–CD200 into the DG region increased the level of anti-inflammatory factors Arg-1, YM-1 and interleukin-4 (IL-4) significantly compared with AAV–GFP group (Additional file 1: Fig. S3B Arg-1, *P* = 0.0494; YM-1, *P* = 0.009; IL-4, *P* = 0.033). On the contrary, although pro-inflammatory factors IL-6 and interleukin-1β (IL-1β) had no obvious changes, tumor necrosis factor-α (TNF-α), another pro-inflammatory cytokine showed an evident decrease in the AAV–CD200 group after CSDS (Additional file 1: Fig. S3B TNF-α, *P* = 0.030; IL-6, *P* = 0.244; IL-1β, *P* = 0.691).

These results suggest that chronic stress induces the activation of microglia and the release of pro-inflammatory cytokines in the DG brain region, while CD200 inhibits microglia hyperactivation and hippocampal neuroinflammation, which may relate to the antidepressant effect of CD200.

### CD200 relieves the impairment of adult hippocampal neurogenesis in susceptible mice

Recent studies have found that Arg-1^+^ microglia in the DG restore hippocampal neurogenesis and enhance the resilience of mice to stress [17]. Therefore, we detected the changes in BDNF and neurogenesis in susceptible mice after injection of CD200Fc in the DG region (Additional file 1: Fig. S4A). Immunofluorescence results showed that after CSDS, the fluorescence intensity of BDNF in the DG region was decreased compared with that of control mice, while it was recovered after CD200Fc injection (Additional file 1: Fig. S4B, C Stress Factor: *F*_(1, 32)_ = 16.59, *P* < 0.001). To examine the proliferation of the neuroblasts in DG of susceptible mice, BrdU was injected (Additional file 1: Fig. S5A) to label newly divided cells and DCX was used as a marker of neuroblasts [44] (Additional file 1: Fig. S5B). The results demonstrated that CSDS reduced the numbers of both BrdU^+^ and BrdU^+^ DCX^+^ cells in DG, and the effect was prevented by CD200Fc administration (Additional file 1: Fig. S5C Stress Factor *F*_(1, 11)_ = 137.7, *P* < 0.001; Fig. S5D Stress Factor: *F*_(1, 11)_ = 47.83, *P* < 0.001,), indicating that CSDS suppresses the proliferation of neuroblasts and CD200Fc can alleviate the impairment of adult hippocampal neurogenesis in stressed mice.

**Fig. 6:**
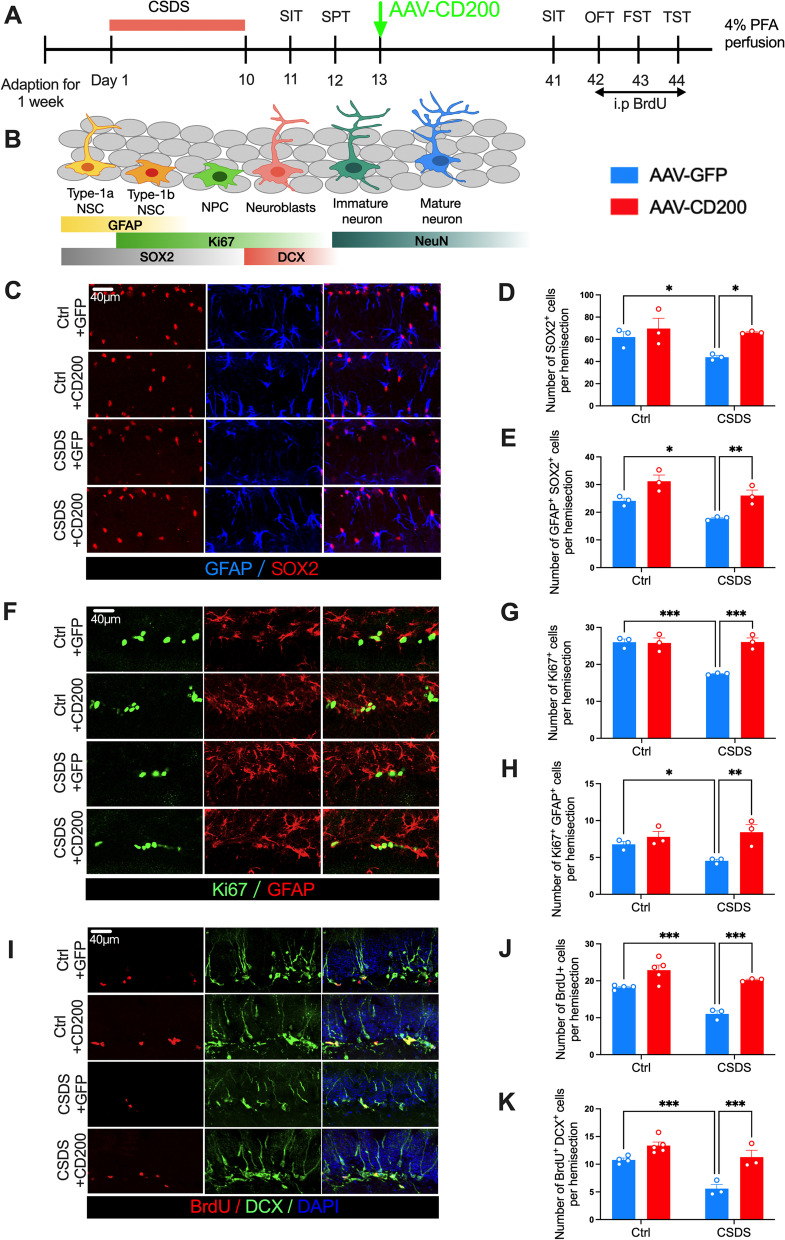
CD200Fc alleviates adult hippocampal neurogenesis impairment induced by CSDS. **A** Experimental timelines for CSDS procedure, AAV–CD200 injection and behavioral testing. **B** Hippocampal neurogenesis process, including cellular types and their specific markers. **C** Representative immunostaining for GFAP (blue)/SOX2 (red) in the DG of control and CSDS group with or without AAV–CD200. Scale bars: 40 μm. **D**, **E** Statistical graph for the numbers of GFAP^+^ (**D**) and GFAP^+^ SOX2^+^ (**E**) cells in the DG of control and CSDS group with or without AAV–CD200 (*n* = 3 mice per group, two-way ANOVA with Fisher’s LSD test). **F** Representative immunostaining for Ki67 (green)/GFAP (red) in the DG of control and CSDS group with or without AAV–CD200. Scale bars: 40 μm. **G**, **H** Statistical graph for the numbers of Ki67^+^ (**G**) and Ki67^+^ GFAP^+^ (**H**) cells in the DG of control and CSDS group with or without AAV–CD200 (*n* = 3 mice per group, two-way ANOVA with Fisher’s LSD test). **I** Representative immunostaining for BrdU (red)/DCX (green)/DAPI (blue) in the DG of control and CSDS group with or without AAV–CD200. Scale bars: 40 μm. **J**, **K** Statistical graph for the number of BrdU^+^ (**J**) and BrdU^+^ DCX^+^ (**K**) cells in the DG of control and CSDS group with or without AAV–CD200 (*n* = 3–5 mice per group, two-way ANOVA with Fisher’s LSD test). Data are expressed as mean ± SEM. *P < 0.05; **P < 0.01; ***P < 0.001. Ctrl, control. See Additional file 1: Table S2 for detailed statistical information

To further confirm the effects of CD200 on neurogenesis, the AAV–CD200 was injected stereotactically into the DG (Fig. 6A). According to a current model of the adult hippocampal neuronal lineage, a series of molecules can be used to identify each stage [45, 46]. Type 1a (quiescent) and 1b (active) neural stem cells (NSCs) share some features of astroglia and express GFAP and SOX2. Type 1b NSCs, neural progenitor cells (NPCs), and neuroblasts are proliferative cells and express Ki67 [47] (Fig. 6B). First, the numbers of SOX2^+^ and GFAP^+^ SOX2^+^ cells in DG (Fig. 6C) were found to be decreased in susceptible mice compared with control mice, while these cells were increased following injection of AAV–CD200 (CSDS Factor: *F*_(1, 8)_ = 7.882, *P* = 0.023, Fig. 6D; CSDS Factor: *F*_(1, 8)_ = 24.03, *P* = 0.001, Fig. 6E). Then, the proportion of Ki67^+^ and Ki67^+^ GFAP^+^ cells were quantified in control and stressed mice (Fig. 6F). The results showed that CSDS reduced the numbers of Ki67^+^ and Ki67^+^/GFAP^+^ cells in DG, whereas this effect was suppressed by AAV–CD200 administration (*F*_(1, 8)_ = 20.84, *P* = 0.002, Fig. 6G; CSDS Factor: *F*_(1, 8)_ = 13.58, *P* = 0.006, Fig. 6H). Similarly, infusion of AAV–CD200 reversed CSDS-induced reduction in the numbers of BrdU^+^ and BrdU^+^ DCX^+^ cells in DG (Fig. 6I; CD200 Factor: *F*_(1, 11)_ = 22.60, *P* < 0.001, Fig. 6J; CD200 Factor: *F*_(1, 11)_ = 23.47, *P* < 0.001, Fig. 6K).

The above results indicate that CD200 increases the number of NSCs, including activated NSCs in the DG region of mice after exposure to CSDS. The number of neuroblasts in the subsequent neurogenesis process was also increased. Thus, it is suggested that upregulation of CD200 in DG ameliorates neurogenesis impairment in the adult hippocampus of stressed mice.

## Discussion

In the present study, we provided the evidence that CD200 exerted an antidepressant action in the mouse model of depression. Overexpression of CD200 in the DG region relieved social avoidance and despair behaviors of stressed mice, whereas knockdown of CD200 in DG facilitated susceptibility to the stress. As an important factor to maintain microglial homeostasis, the antidepressant effect of CD200 was mediated by the receptor CD200R1, which relieved microglia hyperactivation and hippocampal neuroinflammation. Then, CD200 ameliorated the impairment of adult hippocampal neurogenesis induced by CSDS, which was related to the increase in BDNF level in the DG region. Thus, CD200 may serve as a potential target for the therapy of depression.

Previous studies have reported that chronic unpredictable mild stress decreased CD200 mRNA levels in DG [15]. The downregulation of CD200 protein level has also been reported in the prefrontal cortex of female rats in the ovariectomy combined with a chronic unpredictable stress model [48]. In our present study, through screening of brain regions related to stress and emotion, both mRNA and protein level of CD200 were found to be downregulated in the DG region after exposure to CSDS, which was consistent with previous reports. Previous studies observed the changes in CD200 during stress, but the exact effect of CD200 on depression is not clear. To address this issue, we identified the role of CD200 in pathophysiology of MDD. First, after the recombinant protein of CD200 and AAV–CD200 were given into the DG to upregulate CD200 expression, the depressive-like behavior of stressed mice was significantly improved. Second, when the CD200 gene in the DG brain region was silenced and subthreshold stress was applied, the results showed that the knockdown of CD200 in the DG region increased the susceptibility of mice to stress. Therefore, CD200 in the DG region has antidepressant effect.

As the cognate receptor of CD200 [41], CD200R1 is crucial for the ligand–receptor signaling, and CD200–CD200R1 signaling is known to be involved in neuroinflammation of CNS disorders. Reduced expressions of CD200 and CD200R1 have been observed in the brains of patients with multiple sclerosis [49] and Alzheimer's disease [50]. Monocyte-derived macrophages from individuals with Parkinson’s disease show altered CD200R1 regulation in response to inflammatory stimulus [51]. In addition, previous studies have reported the role of CD200–CD200R1 signaling in stress. Exposure to an acute stressor with inescapable tail-shock disrupts CD200–CD200R1 signaling, and treatment with mCD200Fc blocks the stress-induced microglia priming [52, 53]. A decrease expression of CD200R1 has also been observed in the hippocampus in a rat model of early life social isolation [54]. Therefore, the effect of CD200-CD200R1 signaling on chronic stress was researched here. Using LV-siCD200R1 to knockdown CD200R1 in the DG region, we found that CD200Fc administration did not improve depressive-like behaviors induced by CSDS, indicating that the antidepressant effect of CD200 is mediated by its receptor CD200R1. In the CNS, CD200R1 receptor is expressed in microglia [22], and our immunofluorescence staining also confirmed the distribution. Actually, microglia play an important role in healthy and disordered brains, regulating synaptic transmission and neuronal plasticity [55, 56]. Under physiological conditions, microglia monitor the local microenvironmental changes in the CNS constantly. When tissue injury, infections or other pathological processes occur in the brain, microglia are rapidly activated and respond to the challenges. Hyperactive microglia undergo additional morphological change and exhibit high levels of inflammatory factors, eventually leading to cognitive and emotional disorders [57, 58]. A large number of studies have reported that microglial activation plays a role in different depressive models, including CSDS [59], chronic unpredictable mild stress, lipopolysaccharide [60, 61] and social separation-induced depressive-like behaviors [54], and researchers even believe that depression is a microglia-associated disease [62]. Thus, regulation of the activation of microglia may become a new direction for the treatment of depression.

CD200 and CD200R1 interaction has been reported to participate in modulating microglial activation [24], so microglial status was investigated here using exogenous CD200Fc. Results of immunofluorescence showed hyperactivation of microglia during CSDS, while exogenous CD200Fc treatment in the DG significantly alleviated the excessive activation of microglia. This further proves that the activation of the CD200–CD200R1 pathway is crucial for maintaining the resting state of microglia in the CSDS model. Microglia have phenotypic plasticity and can be stimulated by various cytokines to regulate physiological responses and behavioral outcomes during stress [63]. We observed that the fluorescence intensity of CD68, a marker of activated microglia was decreased, and Arg-1, a marker of microglia with a non-inflammatory phenotype was increased within the microglial after CD200 supplementation under the stress. Meanwhile, the decreased gene expression of pro-inflammatory cytokines and increased gene expression of anti-inflammatory cytokines were also observed in stressed mice with AAV–CD200 injection into the DG region. These results demonstrate the capability of CD200 to alleviate microglia activation and hippocampal inflammation. However, the activation phenotype of microglia is complex, which is classified by a different set of surface antigens, metabolic pathways and functions [64]. To better elucidate the activation state of microglia, more specific technique, such as single-cell RNA sequencing or cytometry by time-of-flight mass spectrometry, needs to be used to evaluate microglial phenotypes and functions. In addition, since Iba1 is expressed in both microglial and infiltrating macrophages [65], it is difficult to delineate resident microglia from infiltrating myeloid cells. Furthermore, the disruption of blood–brain barrier (BBB) under chronic stress will induce blood-derived monocytes to infiltrate the parenchyma, where they further differentiate into macrophages [66]. Therefore, other infiltrating macrophages may also contribute to the neuroinflammatory response in hippocampu**s**.

The dysfunction of BBB has been reported to amplify neuroinflammation and microglia activation, acting as a key process in the development of neuroinflammation [67]. In contrast, the reduced expression of CD200 and CD200R1 has been observed after intracerebral hemorrhage, and CD200Fc administration could attenuate BBB leakage and improve neurological functions [68]. Thus, the alleviation of BBB disruption may be also responsible for the antidepressant effects of CD200. Besides, CD200 is not only expressed on neurons, it is also expressed on endothelial cells and other cell types [21]. Thus, CD200 may not be solely responsible for the observed effects on neurons and microglia, the impact of CD200 on endothelial cell and angiogenesis needs further research in the future. Notably, an increased number of phagocytic microglia during chronic stress was observed here, identified by CD68 staining [69], which may contribute to the reduction of neuronal activity via pruning of synaptic connections. In contrast, when treated with CD200, the decreased fluorescence intensity of CD68 indicated the reduced number of phagocytic microglia and the improvement of neuronal activity, which may also be one of the reasons for CD200 to alleviate depressive-like behaviors.

It has been reported that microglia can shape adult hippocampal neurogenesis [70], and increasing Arg-1^+^ microglia in the hippocampus enhances hippocampal neurogenesis and protects mice from stress-induced depressive-like behaviors [17], so the effects of CD200 on neurogenesis was further investigated. The elevation of BDNF fluorescence intensity preliminarily suggested that CD200 can promote nerve growth. Correspondingly, with different markers of neurogenesis, overexpression of CD200 was found to not only facilitate the proliferation and differentiation of neuroblasts but also increase the number of activated NSCs. Therefore, we speculate that CD200 may promote neurogenesis by inhibiting microglia hyperexcitation and correspondingly hippocampal neuroinflammation, thereby exerting an antidepressant effect.

## Conclusion

**Fig. 7:**
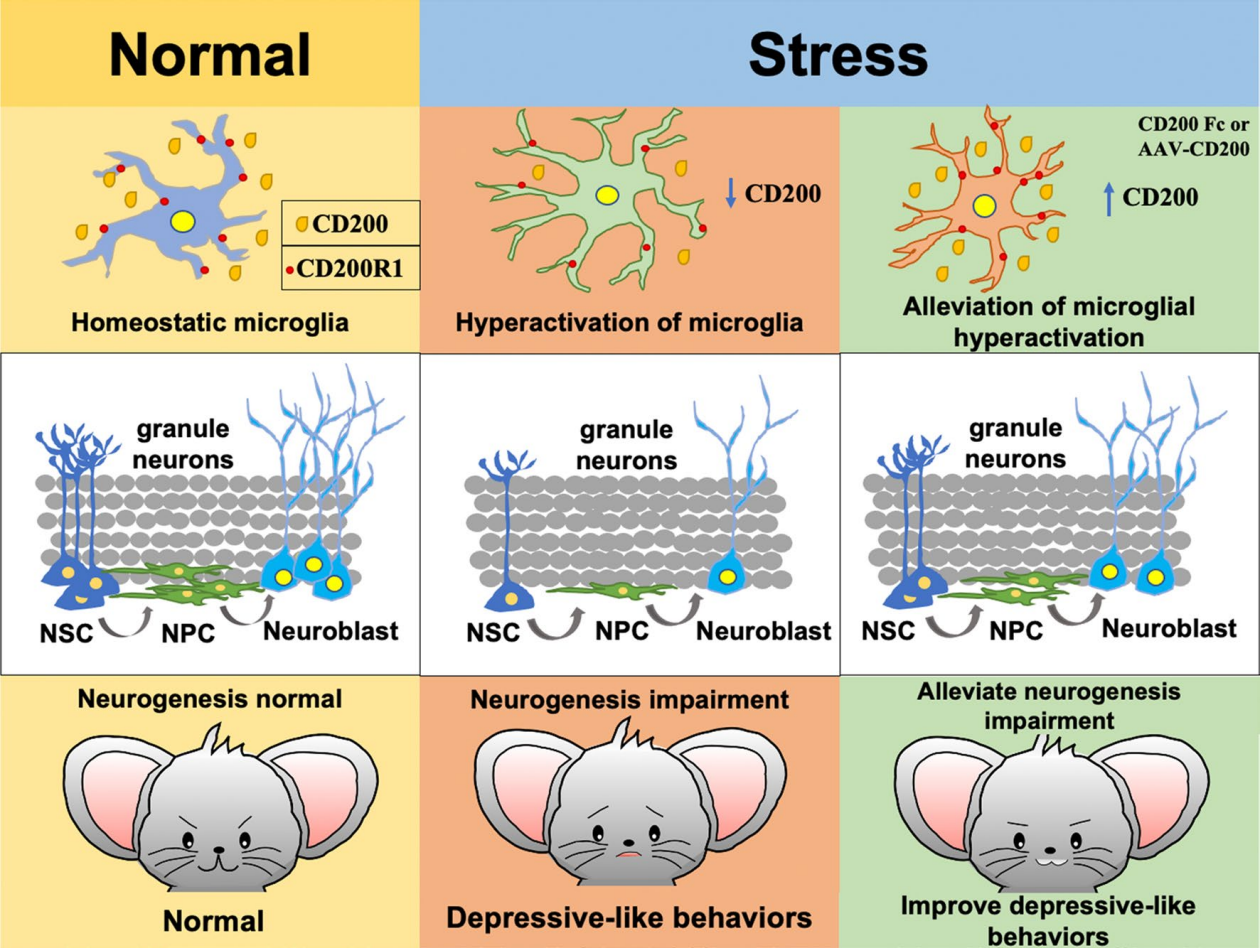
Mechanism diagram for antidepressant effect of CD200 in the dentate gyrus. Under physiological conditions, the interaction of CD200 with CD200R1 maintains microglia in an inactive, homeostatic state. After chronic stress, the reduction of CD200 expression in the DG induces the microglia hyperactivation and then the damage of adult hippocampal neurogenesis, leading to depressive-like behaviors of mice. Upregulation of CD200 levels in the DG inhibits microglia hyperactivation induced by CSDS, and then alleviates adult hippocampal neurogenesis impairment, thereby exerting an antidepressant effect

Our results reveal that CD200 has an antidepressant action in the chronic social defeat stress mouse model, which acts through its receptor. The antidepressant effect of the CD200–CD200R1 pathway may be related to inhibition of microglia hyperactivation and reduction of hippocampal neuroinflammation, which improves the adult hippocampal neurogenesis (Fig. 7). Therefore, developing CD200 mimetics that can cross the BBB or drugs that targeting CD200–CD200R1 signaling would be the direction for clinical translation of CD200 treatment in CNS diseases.

## Supplementary Information

**Additional file 1: Figure S1.** mRNA expression of CD200 in different brain regions after exposure to CSDS. A Analysis of CD200 mRNA expression in the mPFC region of mice after CSDS. B Analysis of CD200 mRNA expression in the Hip region of mice after CSDS. C Analysis of CD200 mRNA expression in the DG region of mice after CSDS. D Analysis of CD200 mRNA expression in the CA1 region of mice after CSDS. E Analysis of CD200 mRNA expression in the CA3 region of mice after CSDS. Data are expressed as mean ± SEM. *P < 0.05; ***P < 0.001. **Figure S2.** Injection of CD200Fc into the lateral ventricle alleviates CSDS-induced depressive-like behavior. A Experimental timelines for CSDS procedure, CD200Fc administration and behavioral testing. B The social interaction ratio in the SIT of control and CSDS mice with exogenous CD200Fc injection. C, D The immobility time in the TSTand FSTof control and CSDS mice with exogenous CD200Fc injection. E The total distance in the OFT of control and CSDS mice with exogenous CD200Fc injection. Data are expressed as mean ± SEM. *P < 0.05; **P < 0.01; ***P < 0.001. **Figure S3.** Overexpression of CD200 in DG facilitates the expression of anti-inflammatory factors after CSDS. A Experimental timelines for CSDS procedure, AAV–CD200 stereotaxical injection and behavioral testing. B Analysis of mRNA levels of Arg-1, YM-1, IL-4, TNF-α, IL-6, and IL-1β after injection of AAV–CD200 virus to the DG region in susceptible mice. Data are expressed as mean ± SEM. *P < 0.05; **P < 0.01. **Figure S4.** CD200 increases the expression of BDNF in the DG brain region of susceptible mice. A Experimental timelines for CSDS procedure, CD200Fc administration and behavioral testing. B Representative immunostaining for BDNFin the DG of control and CSDS group after CD200Fc administration. Scale bars: 80 μm. C The fluorescence intensity of BDNF in the DG of control and CSDS group after CD200Fc administration. Data are expressed as mean ± SEM. *P < 0.05; ***P < 0.001. **Figure S5.** CD200Fc ameliorates adult hippocampal neurogenesis impairment in susceptible mice. A Experimental timelines for CSDS procedure, CD200Fc administration and behavioral testing. B Representative immunostaining for BrdU/DCX in the DG of control and CSDS group after CD200Fc administration. Scale bars: 40 μm. C, D Statistical graph for the number of BrdU^+^ and BrdU^+^ DCX^+^ cells in the DG of control and CSDS group after CD200Fc administration. Data are expressed as mean ± SEM. *P < 0.05; **P < 0.01; ***P < 0.001. **Figure S6.** Raw blot data for Fig. 1G, 3C and 4D. **Table S1.** Sequences of primers for qPCR. **Table S2.** Statistical analysis for each figure.

## Data Availability

The data sets used and/or analyzed during the current study are available from the corresponding authors upon request.

## Abbreviations

AAV: Adeno-associated virus
ACSF: Artificial cerebrospinal fluid
Arg-1: Arginase-1
BDNF: Brain-derived neurotrophic factor
BrdU: 5-Bromo-2-deoxyuridine
BSA: Bovine serum albumin
CD200: Cluster of differentiation 200
CD200Fc: CD200 chimera protein fragment crystallizable
CD200R1: CD200 receptor 1
CD68: Cluster of differentiation 68
CSDS: Chronic social defeat stress
DCX: Doublecortin
DG: Dentate gyrus
FST: Forced swimming test
GFAP: Glial fibrillary acidic protein
GFP: Green fluorescent protein
HCl: Hydrochloric acid
HRP: Horseradish peroxidase
Iba1: Ionized calcium binding adaptor molecule 1
IL-1β: Interleukin-1β
IL-4: Interleukin-4
IL-6: Interleukin-6
LV: Lentiviral vectors
MDD: Major depressive disorder
mPFC: Medial prefrontal cortex
NPC: Neural progenitor cell
NSC: Neural stem cell
OFT: Open field test
PBS: Phosphate buffer saline
SDS: Sodium dodecyl sulfate
SIT: Social interaction test
SPT: Sucrose preference test
SSDS: Subthreshold social defeat stress
TNF-α: Tumor necrosis factor-α
TST: Tail suspension test
