# Supplementary material for: CD200 in dentate gyrus improves depressive-like behaviors of mice through enhancing hippocampal neurogenesis via alleviation of microglia hyperactivation
Source: J Neuroinflammation. 2023 Jun 30;20:157. doi: 10.1186/s12974-023-02836-4 (PMC10314496; doi:10.1186/s12974-023-02836-4)
Supplement: Supplementary file 1 — Additional file 1: Figure S1. mRNA expression of CD200 in different brain regions after exposure to CSDS. A Analysis of CD200 mRNA expression in the mPFC region of mice after CSDS. B Analysis of CD200 mRNA expression in the Hip region of mice after CSDS. C Analysis of CD200 mRNA expression in the DG region of mice after CSDS. D Analysis of CD200 mRNA expression in the CA1 region of mice after CSDS. E Analysis of CD200 mRNA expression in the CA3 region of mice after CSDS. Data are expressed as mean ± SEM. *P < 0.05; ***P < 0.001. Figure S2. Injection of CD200Fc into the lateral ventricle alleviates CSDS-induced depressive-like behavior. A Experimental timelines for CSDS procedure, CD200Fc administration and behavioral testing. B The social interaction ratio in the SIT of control and CSDS mice with exogenous CD200Fc injection. C, D The immobility time in the TSTand FSTof control and CSDS mice with exogenous CD200Fc injection. E The total distance in the OFT of control and CSDS mice with exogenous CD200Fc injection. Data are expressed as mean ± SEM. *P < 0.05; **P < 0.01; ***P < 0.001. Figure S3. Overexpression of CD200 in DG facilitates the expression of anti-inflammatory factors after CSDS. A Experimental timelines for CSDS procedure, AAV–CD200 stereotaxical injection and behavioral testing. B Analysis of mRNA levels of Arg-1, YM-1, IL-4, TNF-α, IL-6, and IL-1β after injection of AAV–CD200 virus to the DG region in susceptible mice. Data are expressed as mean ± SEM. *P < 0.05; **P < 0.01. Figure S4. CD200 increases the expression of BDNF in the DG brain region of susceptible mice. A Experimental timelines for CSDS procedure, CD200Fc administration and behavioral testing. B Representative immunostaining for BDNFin the DG of control and CSDS group after CD200Fc administration. Scale bars: 80 μm. C The fluorescence intensity of BDNF in the DG of control and CSDS group after CD200Fc administration. Data are expressed as mean ± SEM. *P < 0.05; ***P < 0.001. Figure S5. CD2 [file 12974_2023_2836_MOESM1_ESM.docx]

**CD200 in dentate gyrus improves depressive-like behaviors of mice through enhancing hippocampal neurogenesis via alleviation of microglia hyperactivation**

Xi Chen^1^, Qian-Qian Cui^1^, Xiao-Hai Hu^1^, Jian Ye^1^, Zi-Cun Liu^1^, Yuan-Xi Mei^1^, Fang Wang^1,2,3,4^, Zhuang-Li Hu^1,2,3,4,*^ and Jian-Guo Chen^1,2,3,4,*^

**^1^**Department of Pharmacology, School of Basic Medicine, Tongji Medical College, Huazhong University of Science and Technology, Wuhan, China. ^2^The Key Laboratory for Drug Target Researches and Pharmacodynamic Evaluation of Hubei Province, Wuhan, China. ^3^The Research Center for Depression, Tongji Medical College, Huazhong University of Science, 430030, Wuhan, China. ^4^Key Laboratory of Neurological Diseases (HUST), Ministry of Education of China, Wuhan, China.

**Running title: CD200 improves depression via alleviation of microglia-mediated neurogenesis impairment**

Correspondence: J.-G. Chen, chenj@mails.tjmu.edu.cn or Z.-L. Hu, zlhu916@hust.edu.cn

**Supplementary Information**

**Materials and methods**

*Real-time quantitative PCR*

Real-time quantitative PCR was performed according to the previous protocol ^1,2^. Total RNA was extracted from tissues using Trizol reagent (Invitrogen, ThermoFisher Scientific Inc, Waltham, MA, USA) and quantified. 1000 ng RNA was used for cDNA synthesis with the instruction of RevertAid First Strand cDNA Synthesis kit (Fermentas, ThermoFisher Scientific Inc, Waltham, Massachusetts, USA). Quantitative real-time PCR was performed in a StepOnePlus^TM^ Real-Time PCR System (Applied Biosystems, Foster City, California, USA) with SYBR Green PCR Master Mix (Applied Biosystems, Foster City, California, USA). 500 ng cDNA was diluted to 200 μl, and 3 μl was used for each reaction. Samples were then heated to 95 °C for 10 min followed by 40 cycles of 95 °C for 15 sec, 60 °C for 30 sec, and 72 °C for 30 sec. Analysis of gene expression was performed using ^ΔΔ^Ct and all samples were normalized to GAPDH. The sequences of the primers are shown in Table S1.

**References**

1. Xie WL, Zheng HL, Li HH, Lu JJ, Xue SG, Luo Y, et al. Deficiency of Glycosylated α-Dystroglycan in Ventral Hippocampus Bridges the Destabilization of Gamma-Aminobutyric Acid Type A Receptors With the Depressive-like Behaviors of Male Mice. Biol Psychiatry. 2021;91:593-603.

2. Cui QQ, Hu ZL, Hu YL, Chen X, Wang J, Mao L, et al. Hippocampal CD 39/ ENTPD 1 promotes mouse depression‐like behavior through hydrolyzing extracellular ATP. EMBO Rep. 2020;21:e47857.

**Supplementary Figures**

**
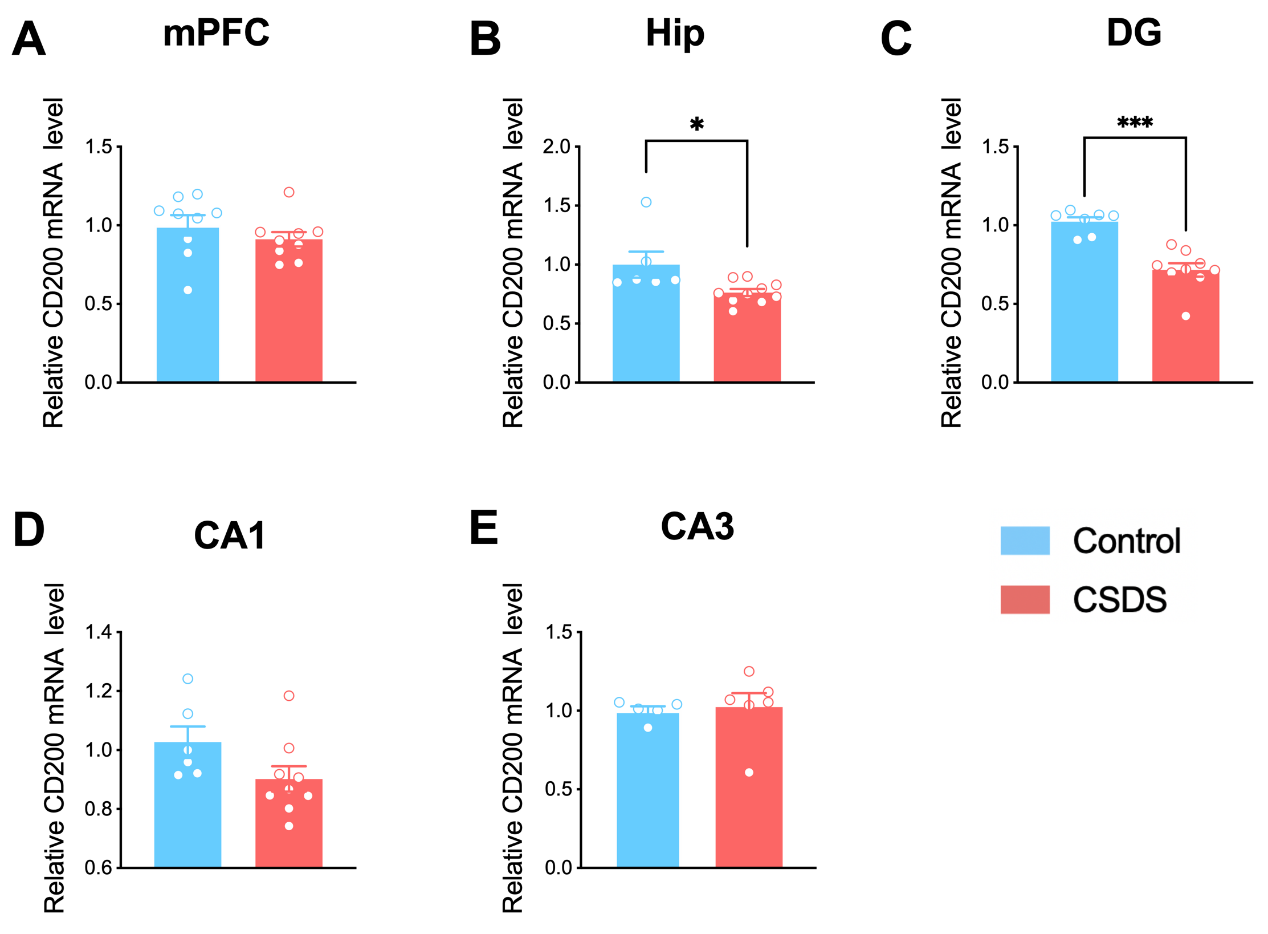
**

**Figure S1. The mRNA expression of CD200 in different brain regions after exposure to CSDS.** **A** Analysis of CD200 mRNA expression in the mPFC region of mice after CSDS (n = 9 per group, Student’s *t* test). **B** Analysis of CD200 mRNA expression in the Hip region of mice after CSDS (n = 6-10 per group, Student’s *t* test). **C** Analysis of CD200 mRNA expression in the DG region of mice after CSDS (n = 7-9 per group, Student’s *t* test). **D** Analysis of CD200 mRNA expression in the CA1 region of mice after CSDS (n = 6-9 per group, Student’s *t* test). **E** Analysis of CD200 mRNA expression in the CA3 region of mice after CSDS (n = 5-6 per group, Student’s *t* test). Data are expressed as mean ± SEM. *P < 0.05; ***P < 0.001. Hip, hippocampus; DG, dentate gyrus. See Table S2 for detailed statistical information.

**
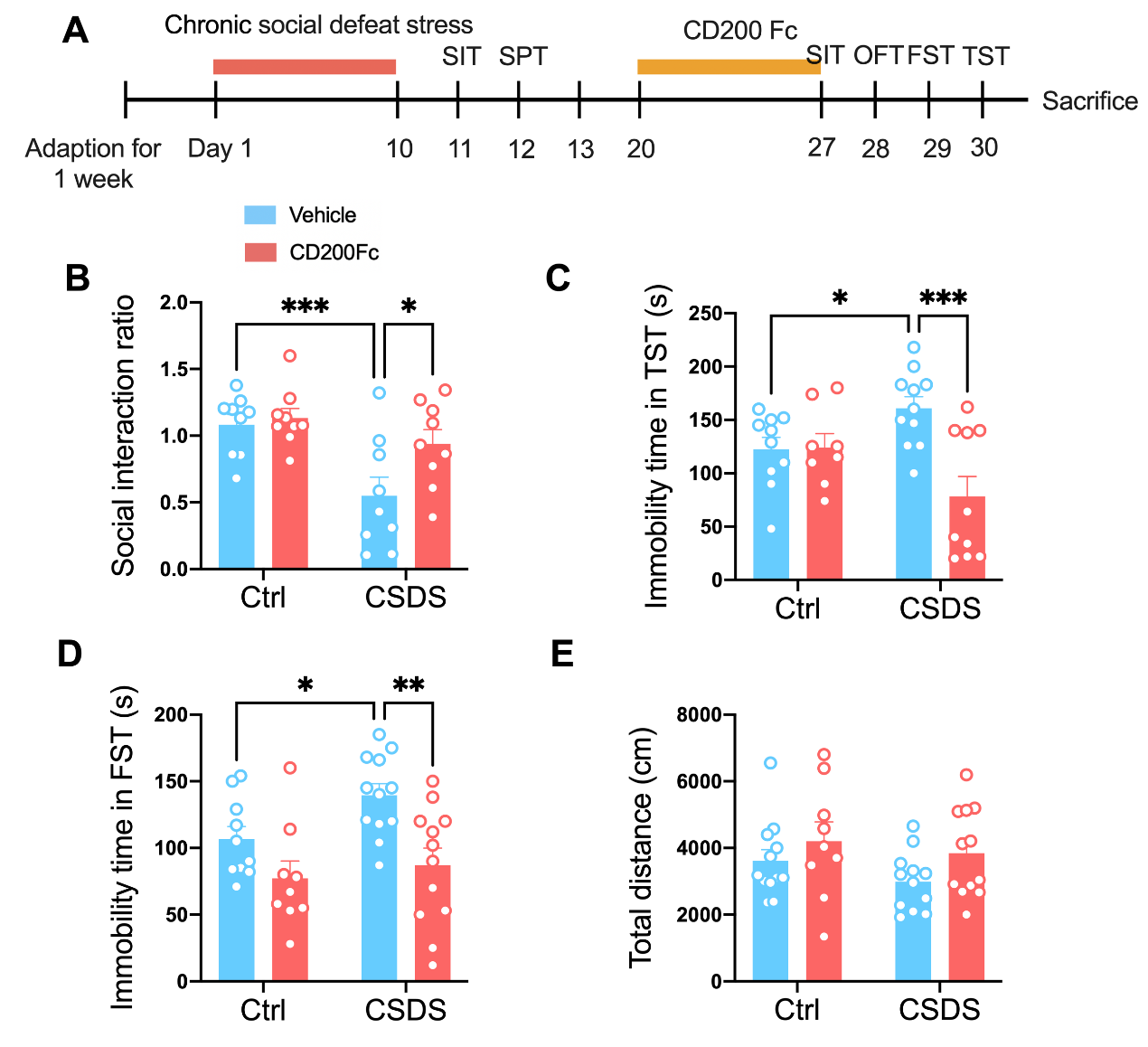
**

**Figure S2. Injection of CD200Fc into the lateral ventricle alleviates CSDS-induced depressive-like behavior.** **A** Experimental timelines for CSDS procedure, CD200Fc administration and behavioral testing. **B** The social interaction ratio in the SIT of control and CSDS mice with exogenous CD200Fc injection (n = 9 per group, two-way ANOVA with Fisher’s LSD test). **C**-**D** The immobility time in the TST (C) and FST (D) of control and CSDS mice with exogenous CD200Fc injection (TST, n = 8-11 per group; FST, n = 9-12 per group, two-way ANOVA with Fisher’s LSD test). **E** The total distance in the OFT of control and CSDS mice with exogenous CD200Fc injection (n = 9-12 per group, two-way ANOVA with Fisher’s LSD test). Data are expressed as mean ± SEM. *P < 0.05; **P < 0.01; ***P < 0.001. Ctrl, control. See Table S2 for detailed statistical information.

**
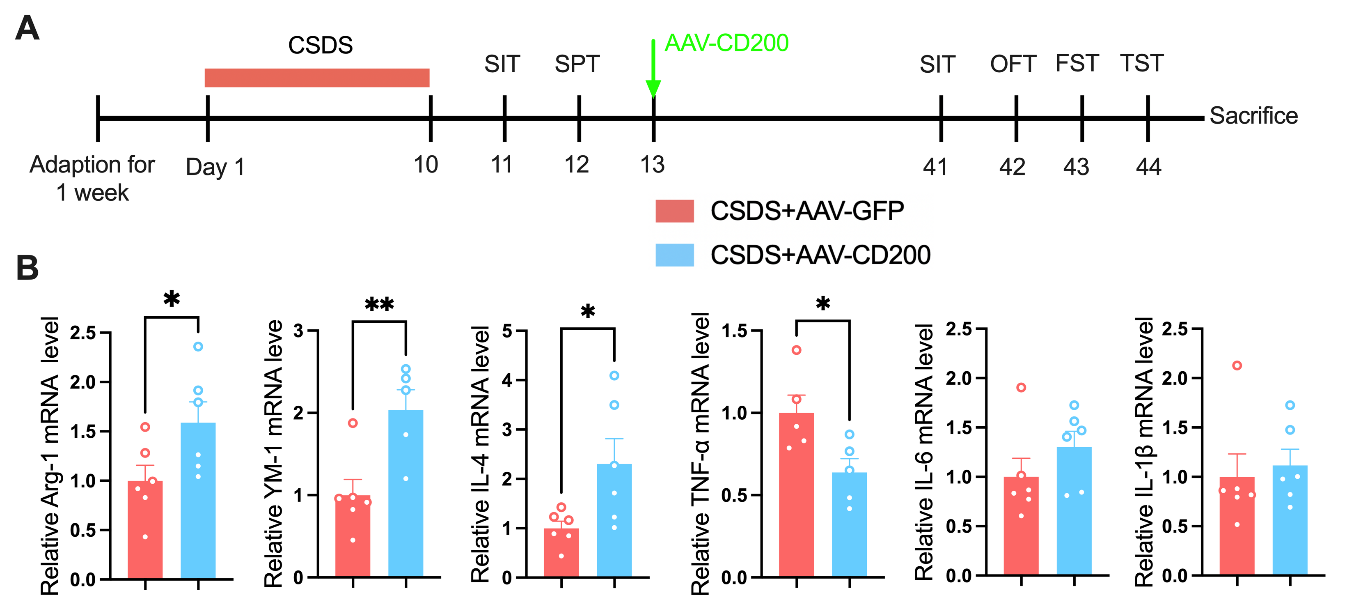
**

**Figure S3. Overexpression of CD200 in DG facilitates the expression of anti-inflammatory factors after CSDS.** **A** Experimental timelines for CSDS procedure, AAV-CD200 stereotaxical injection and behavioral testing. **B** Analysis of mRNA levels of Arg-1, YM-1, IL-4, TNF-α, IL-6, and IL-1β after injection of AAV-CD200 virus to the DG region in susceptible mice (Arg-1 group, n = 6 per group; YM-1 group, CSDS + AAV-GFP: n = 5-6 per group; IL-4 group, n = 6 per group; TNF-α group, n = 5 per group; IL-6 group, n = 6 per group; IL-1β group, n = 6 per group; Student’s *t* test). Data are expressed as mean ± SEM. *P < 0.05; **P < 0.01. See Table S2 for detailed statistical information.


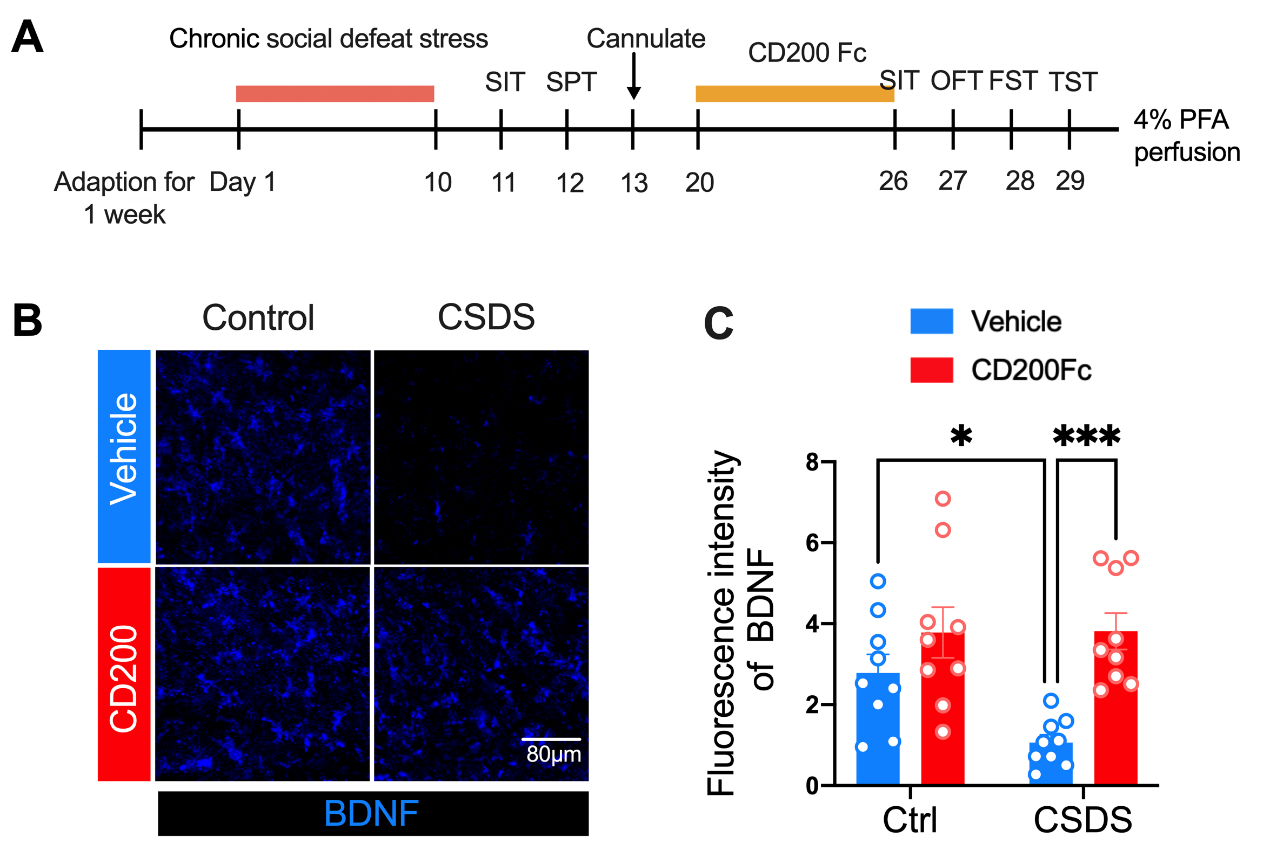


**Figure S4. CD200 increases the expression of BDNF in the DG brain region of susceptible mice. A** Experimental timelines for CSDS procedure, CD200Fc administration and behavioral testing. **B** Representative immunostaining for BDNF (blue) in the DG of control and CSDS group after CD200Fc administration. Scale bars: 80 μm. **C** The fluorescence intensity of BDNF in the DG of control and CSDS group after CD200Fc administration (n = 9 sections/group from 3 mice, two-way ANOVA with Fisher’s LSD test). Data are expressed as mean ± SEM. *P < 0.05; ***P < 0.001. Ctrl, control. See Table S2 for detailed statistical information.

**
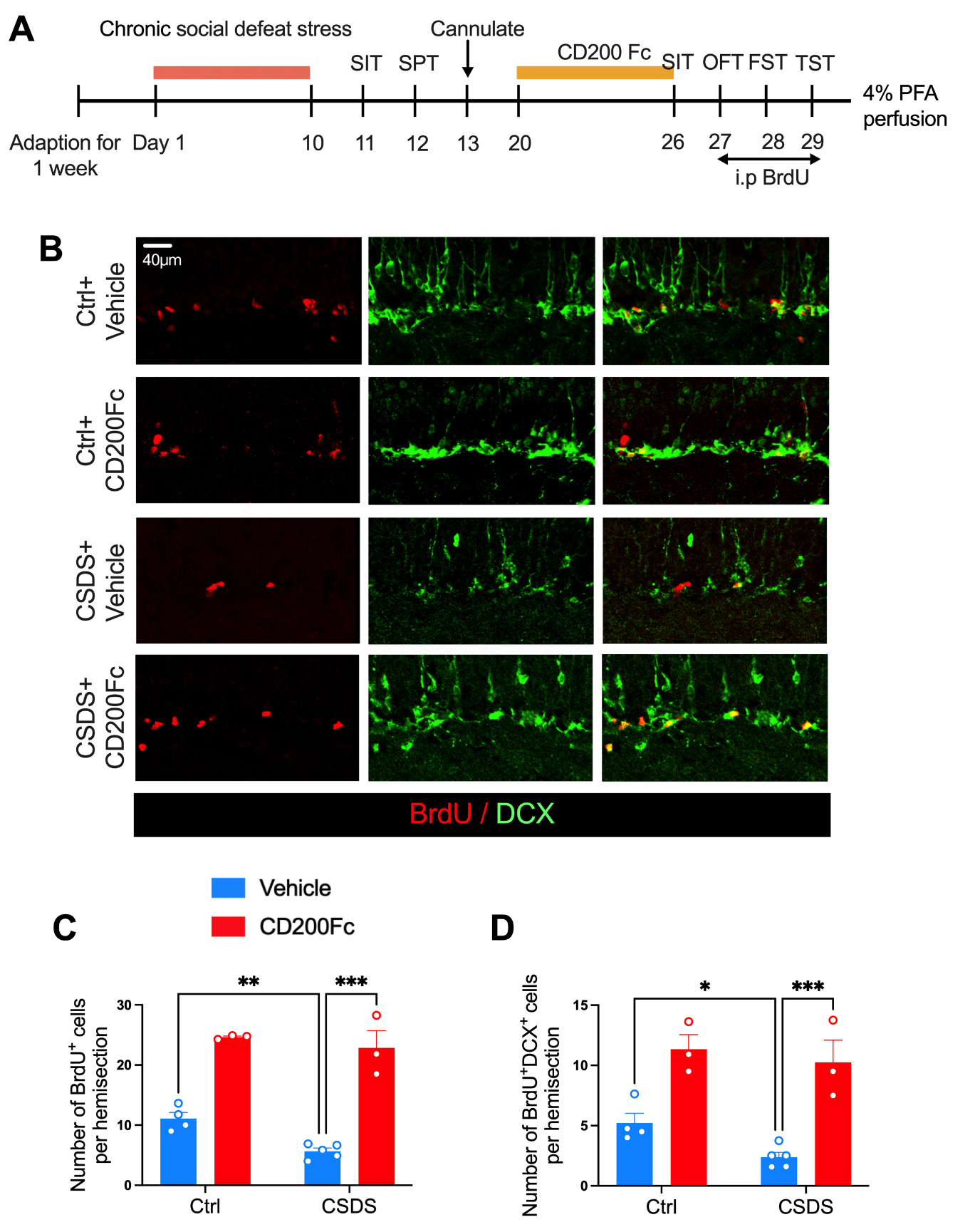
**

**Figure S5. CD200Fc ameliorates adult hippocampal neurogenesis impairment in susceptible mice. A** Experimental timelines for CSDS procedure, CD200Fc administration and behavioral testing. **B** Representative immunostaining for BrdU (red)/DCX (green) in the DG of control and CSDS group after CD200Fc administration. Scale bars: 40 μm. **C**-**D** Statistical graph for the number of BrdU^+^ (C) and BrdU^+^ DCX^+^ (D) cells in the DG of control and CSDS group after CD200Fc administration (n = 3-5 per group, two-way ANOVA with Fisher’s LSD test). Data are expressed as mean ± SEM. *P < 0.05; **P < 0.01; ***P < 0.001. Ctrl: control. See Table S2 for detailed statistical information.


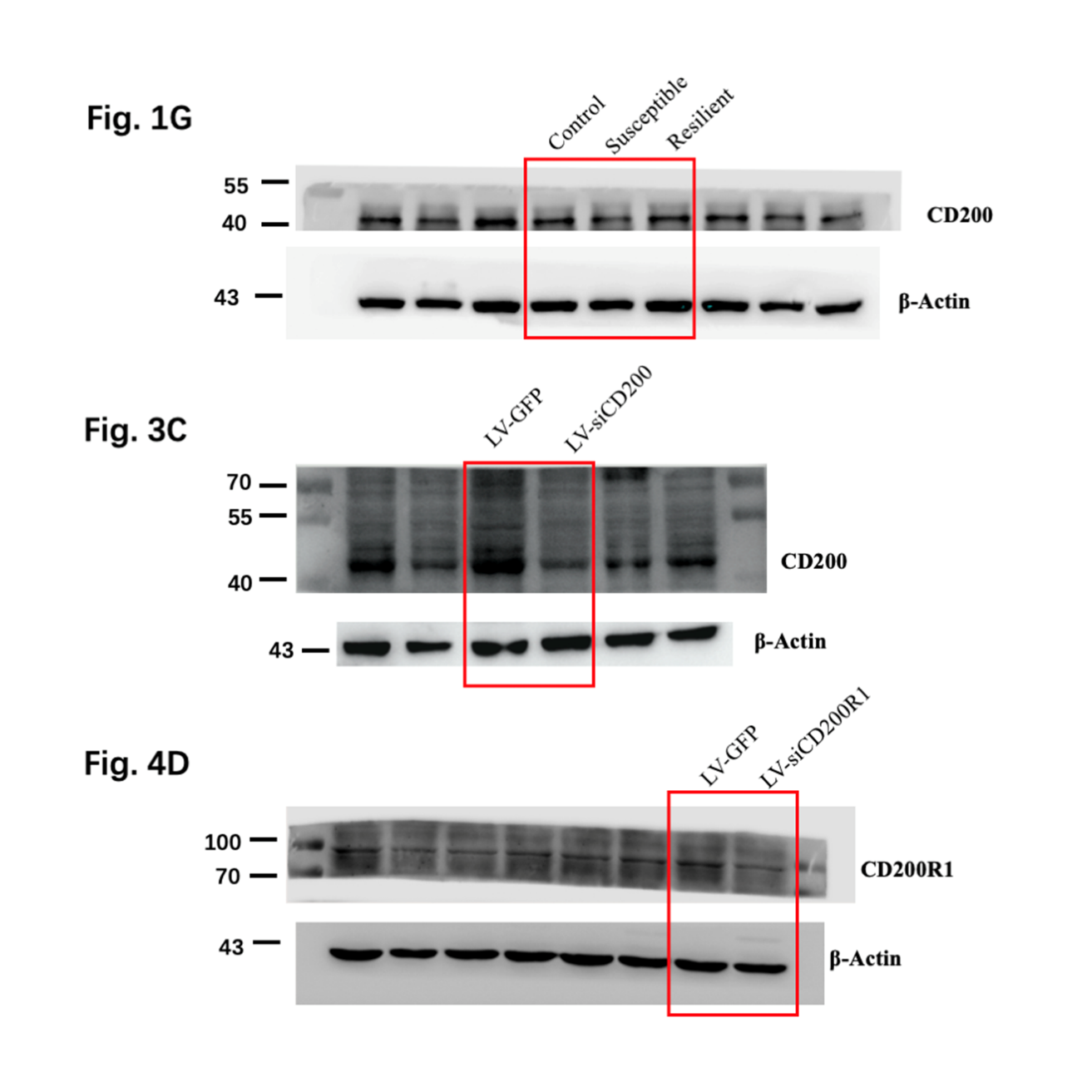


**Figure S6. Raw blot data for Fig. 1G, 3C and 4D.**

**Table S1. The sequences of primers for qPCR**

| Gene Name | Forward (5’-3’) | Reverse (5’-3’) |
| --- | --- | --- |
| CD200 | TGGAGATGGTGAAAGAGGTG | GGTGTTGAAGAGCAGAAAGC |
| Arg-1 | CTCCAAGCCAAAGTCCTTAGAG | AGGAGCTGTCATTAGGGACATC |
| YM-1 | AGAAGGGAGTTTCAAACCTGGT | CTCTTGCTGATGTGTGTAAGTGA |
| IL-4 | ATGGATGTGCCAAACGTCCT | AAGCACCTTGGAAGCCCTAC |
| TNF-α | GTCTACTGAACTTCGGGGTGAT | CTGAGTGTGAGGGTCTGGGC |
| IL-1β | TTGCTTCCAAGCCCTTGACT | CTCCACGGGCAAGACATAGG |
| IL-6 | GGGACTGATGCTGGTGACAA | ACAGGTCTGTTGGGAGTGGT |
| GAPDH | ATGGTGAAGGTCGGTGTG | CATTCTCGGCCTTGACTG |

**Table S2. Statistical analysis for each figure**

| **Figure and numbers of animals used** | **Statistical analysis** | **Post hoc tests** | **Mean ± SEM** |
| --- | --- | --- | --- |
| 1C Control = 12 Susceptible = 11 Resilient = 11 | One-way ANOVA Treatment Factor: F (2, 31) = 36.49/P<0.001 | Fisher's LSD post-test Control vs. Susceptible/ P<0.001 Susceptible vs. Resilient/ P<0.001 | Control = 1.365 ± 0.06510 Susceptible = 0.6506 ± 0.06823 Resilient = 1.230 ± 0.05288 |
| 1D Control (No Target) = 12 Control (Target) = 12 Susceptible (No Target) = 11 Susceptible (Target) = 11 Resilient (No Target) = 12 Resilient (Target) = 12 | Two-way ANOVA Interaction Factor: F (2, 64) = 10.83/P<0.001 Stress Factor: F (1, 64) = 2.244/P=0.193 Target Factor: F (2, 62) = 4.150/P=0.020 | Fisher's LSD post-test Target: Control vs. Target: Susceptible P<0.001 | Control (No Target) = 44.03 ± 2.702 Control (Target) = 60.29 ± 4.990 Susceptible (No Target) = 52.25 ± 4.070 Susceptible (Target) = 35.00 ± 4.665 Resilient (No Target) = 47.09 ± 2.208 Resilient (Target) = 63.04 ± 4.988 |
| 1E Control = 16 Susceptible = 12 Resilient = 13 | One-way ANOVA Treatment Factor: F(2,38) = 4.377/P=0.019 | Fisher's LSD post-test Control vs. Susceptible/ P=0.006 Susceptible vs. Resilient/ P=0.044 | Control = 82.94 ± 13.37 Susceptible = 133.8 ± 11.95 Resilient = 95.46 ± 10.95 |
| 1F Control = 12 Susceptible = 11 Resilient = 12 | One-way ANOVA Treatment Factor: F (2, 32) = 35.51/P<0.001 | Fisher's LSD post-test Control vs. Susceptible/ P<0.001 Susceptible vs. Resilient/ P<0.001 | Control = 91.49 ± 1.768 Susceptible = 74.17 ± 1.542 Resilient = 86.17 ± 1.003 |
| 1G Control = 8 Susceptible = 8 Resilient = 5 | One-way ANOVA Treatment Factor: F (2, 18) = 5.339/P=0.015 | Fisher's LSD post-test Control vs. Susceptible/ P=0.008 Susceptible vs. Resilient/ P=0.021 | Control = 1.000 ± 0.08471 Susceptible = 0.6616 ± 0.09058 Resilient = 0.9915 ± 0.06180 |
| 2B Ctrl+ Vehicle = 9  Ctrl + CD200Fc = 10 CSDS + Vehicle = 12  CSDS + CD200Fc = 10 | Two-way ANOVA Interaction Factor: F (1, 37) = 7.891/P=0.008 CD200 Factor: F (1, 37) = 3.030/P=0.090 Stress Factor: F (1, 37) = 2.041/P=0.161 | Fisher's LSD post-test CSDS: Vehicle vs. Ctrl: Vehicle/ P=0.003 CSDS: CD200Fc vs. CSDS: Vehicle/ P=0.004 | Ctrl + Vehicle = 1.277 ± 0.1518 Ctrl + CD200Fc = 1.018 ± 0.09287 CSDS + Vehicle = 0.4218 ± 0.1023 CSDS + CD200Fc = 1.219 ± 0.3240 |
| 2C Ctrl + Vehicle = 10  Ctrl + CD200Fc = 12 CSDS + Vehicle = 11  CSDS + CD200Fc = 11 | Two-way ANOVA Interaction Factor: F (1, 40) = 7.197/P=0.011 CD200 Factor: F (1, 40) = 0.7813/P=0.382 Stress Factor: F (1, 40) = 5.786/P=0.021 | Fisher's LSD post-test CSDS: Vehicle vs. Ctrl: Vehicle/ P=0.018 CSDS: CD200Fc vs. CSDS: Vehicle/ P<0.001 | Ctrl + Vehicle = 128.1 ± 8.049 Ctrl + CD200Fc = 131.2 ± 9.521 CSDS + Vehicle = 167.5 ± 13.55 CSDS + CD200Fc = 111.3 ± 11.91 |
| 2D Ctrl + Vehicle = 8  Ctrl + CD200Fc = 7 CSDS + Vehicle = 8  CSDS + CD200Fc = 8 | Two-way ANOVA Interaction Factor: F (1, 27) = 6.625/P=0.016 CD200 Factor: F (1, 27) = 10.53/P=0.003 Stress Factor: F (1, 27) = 2.594/P=0.119 | Fisher's LSD post-test CSDS: Vehicle vs. Ctrl: Vehicle/ P<0.001 CSDS: CD200Fc vs. CSDS: Vehicle/ P=0.006 | Ctrl + Vehicle = 55.25 ± 14.80 Ctrl + CD200Fc = 68.00 ± 15.70 CSDS + Vehicle = 132.3 ± 10.42 CSDS + CD200Fc = 76.88 ± 11.82 |
| 2E Ctrl + Vehicle = 11  Ctrl + CD200Fc = 11 CSDS + Vehicle = 11  CSDS + CD200Fc = 11 | Two-way ANOVA Interaction Factor: F (1, 40) = 0.2664/P=0.609 CD200 Factor: F (1, 40) = 0.09396/P=0.761 Stress Factor: F (1, 40) = 0.6154/P=0.437 | Fisher's LSD post-test CSDS: Vehicle vs. Ctrl: Vehicle/ P=0.564 CSDS: CD200Fc vs. CSDS: Vehicle/ P=0.850 | Ctrl + Vehicle = 4196 ± 549.7 Ctrl + CD200Fc = 4708 ± 254.9 CSDS + Vehicle = 4520 ± 251.1 CSDS + CD200Fc = 4625 ± 433.7 |
| 2H AAV-GFP = 6  AAV-CD200 = 6 | AAV-GFP vs. AAV-CD200 /t-test/ p<0.001 |  | AAV-GFP = 1.000 ± 0.07767 AAV-CD200 = 60.13 ± 12.63 |
| 2I Ctr l+ AAV-GFP = 8  Ctrl + AAV-CD200 = 8 CSDS + AAV-GFP = 9  CSDS + AAV-CD200 = 9 | Two-way ANOVA Interaction Factor: F (1, 30) = 1.479/P=0.223 CD200 Factor: F (1, 30) = 14.14/P<0.001 Stress Factor: F (1, 30) = 6.351/P=0.017 | Fisher's LSD post-test Ctrl: AAV-GFP vs. CSDS: AAV-GFP/ P=0.001 CSDS: AAV-GFP vs. CSDS: AAV-CD200/ P=0.011 | Ctrl + AAV-GFP = 1.326 ± 0.1793 Ctrl + AAV-CD200 = 1.542 ± 0.2255 CSDS + AAV-GFP = 0.5022 ± 0.1245 CSDS + AAV-CD200 = 1.121 ± 0.1295 |
| 2J Ctrl + AAV-GFP = 9  Ctrl + AAV-CD200 = 9 CSDS + AAV-GFP = 9  CSDS + AAV-CD200 = 9 | Two-way ANOVA Interaction Factor: F (1, 32) = 5.388/P=0.027 CD200 Factor: F (1, 32) = 14.51/P<0.001 Stress Factor: F (1, 32) = 10.42/P=0.003 | Fisher's LSD post-test Ctrl: AAV-GFP vs. CSDS: AAV-GFP/ P<0.001 CSDS: AAV-GFP vs. CSDS: AAV-CD200/ P<0.001 | Ctrl + AAV-GFP = 104.2 ± 11.02 Ctrl + AAV-CD200 = 91.43 ± 15.40 CSDS + AAV-GFP = 190.8 ± 8.317 CSDS + AAV-CD200 = 112.4 ± 19.23 |
| 2K Ctrl + AAV-GFP = 9  Ctrl + AAV-CD200 = 9 CSDS + AAV-GFP = 10  CSDS + AAV-CD200 = 11 | Two-way ANOVA Interaction Factor: F (1, 35) = 10.54/P=0.003 CD200 Factor: F (1, 35) = 15.76/P<0.001 Stress Factor: F (1, 35) = 3.263/P=0.079 | Fisher's LSD post-test Ctrl: AAV-GFP vs. CSDS: AAV-GFP/ P<0.001 CSDS: AAV-GFP vs. CSDS: AAV-CD200/ P<0.001 | Ctrl + AAV-GFP = 66.02 ± 14.38 Ctrl + AAV-CD200 = 85.71 ± 7.244 CSDS + AAV-GFP = 164.6 ± 16.59 CSDS + AAV-CD200 = 95.59 ± 13.43 |
| 2L Ctrl + AAV-GFP = 9  Ctrl + AAV-CD200 = 9 CSDS + AAV-GFP = 10  CSDS + AAV-CD200 = 11 | Two-way ANOVA Interaction Factor: F (1, 35) = 0.3828/P=0.540 CD200 Factor: F (1, 35) = 1.866/P=0.181 Stress Factor: F (1, 35) = 0.08143/P=0.777 | Fisher's LSD post-test Ctrl: AAV-GFP vs. CSDS: AAV-GFP/ P=0.174 CSDS: AAV-GFP vs. CSDS: AAV-CD200/ P=0.510 | Ctrl + AAV-GFP = 4210 ± 271.5 Ctrl + AAV-CD200 = 4298 ± 144.5 CSDS + AAV-GFP = 4731 ± 332.9 CSDS + AAV-CD200 = 4494 ± 242.7 |
| 3C LV-GFP = 3 LV-siCD200 = 3 | LV-GFP vs. LV-siCD200 /t-test/ p=0.046 |  | LV-GFP = 1.032 ± 0.1250 LV-siCD200 = 0.5614 ± 0.1079 |
| 3E LV-GFP (No Target) = 14 LV-siCD200 (No Target) = 14 LV-GFP-SSDS (No Target) = 12 LV-siCD200-SSDS (No Target) = 13 LV-GFP (Target) = 14 LV-siCD200 (Target) = 14 LV-GFP-SSDS (Target) = 12 LV-siCD200-SSDS(Target) = 13 | One-way ANOVA Treatment Factor: F (7, 98) = 8.126/P<0.001 | Fisher's LSD post-test LV-GFP-SSDS (No Target) vs. LV-siCD200-SSDS (No Target) P=0.171 LV-GFP-SSDS (Target) vs. LV-siCD200-SSDS (Target) p=0.010 | LV-GFP (No Target) = 41.76 ± 3.407 LV-siCD200 (No Target) = 45.96 ± 2.163 LV-GFP-SSDS (No Target) = 36.62 ± 4.559 LV-siCD200-SSDS (No Target) = 45.12 ± 4.046 LV-GFP (Target) = 67.31 ± 4.690 LV-siCD200 (Target) = 66.66 ± 5.168 LV-GFP-SSDS (Target) = 55.98 ± 4.914 LV-siCD200-SSDS(Target) = 39.68 ± 4.381 |
| 3F LV-GFP = 13 LV-GFP-SSDS = 13 LV-siCD200 = 15  LV-siCD200-SSDS= 16 | Two-way ANOVA Interaction Factor: F (1, 53) = 2.521/P=0.118 LV-siCD200 Factor: F (1, 53) = 11.01/P=0.002 SSDS Factor: F (1, 53) = 7.794/P=0.007 | Fisher's LSD post-test SSDS:LV-GFP vs. SSDS:LV-siCD200/ P=0.003 | LV-GFP = 99.31 ± 11.84 LV-GFP-SSDS = 122.6 ± 14.33 LV-siCD200 = 115.5 ± 14.39 LV-siCD200-SSDS= 181.6 ± 12.68 |
| 4D LV-GFP = 3 LV-siCD200R1 = 3 | LV-GFP vs. LV-siCD200R1 /t-test/ p<0.001 |  | LV-GFP = 1.000 ± 0.01961 LV-siCD200R1 = 0.4674 ± 0.04496 |
| 4E Ctrl + LV-GFP = 9 CSDS + LV-GFP = 7 Ctrl + LV-siCD200R1 = 9  CSDS + LV-siCD200R1 = 9 | Two-way ANOVA Interaction Factor: F (1, 30) = 0.1016/P=0.752 LV-siCD200R1 Factor: F (1, 30) = 25.73/P<0.001 CSDS Factor: F (1, 30) = 3.155/P=0.086 | Fisher's LSD post-test  Ctrl: LV-GFP vs. Ctrl: LV-siCD200R1/ P=0.295  CSDS: LV-GFP vs. CSDS: LV-siCD200R1/ P=0.162 Ctrl: LV-GFP vs. CSDS: LV-GFP/ P=0.003  Ctrl: LV-siCD200R1 vs. CSDS: LV-siCD200R1/ p<0.001 | Ctrl + LV-GFP = 1.291 ± 0.1405 CSDS + LV-GFP = 0.7637 ± 0.03780 Ctrl + LV-siCD200R1 = 1.129 ± 0.1211  CSDS + LV-siCD200R1 = 0.5313 ± 0.08855 |
| 4F Ctrl + LV-GFP + Vehicle = 6 Ctrl + LV-siCD200R1+Vehicle = 7 CSDS + LV-GFP + Vehicle = 10 CSDS + LV-siCD200R1 + Vehicle = 11 Ctrl + LV-GFP+CD200Fc = 10 Ctrl + LV-siCD200R1 + CD200Fc = 9 CSDS + LV-GFP + CD200Fc = 7 CSDS + LV-siCD200R1 + CD200Fc = 9 | One-way ANOVA Treatment Factor: F (7, 61) = 11.42/P<0.001 | Fisher's LSD post-test Ctrl + LV-GFP + Vehicle vs. CSDS + LV-GFP + Vehicle P<0.001 Ctrl+LV-siCD200R1+Vehicle vs. CSDS + LV-siCD200R1 + Vehicle P<0.001 Ctrl + LV-GFP + CD200Fc vs. CSDS + LV-GFP + CD200Fc P=0.864 Ctrl + LV-siCD200R1 + CD200Fc vs. CSDS+LV-siCD200R1+CD200Fc P=0.003 | Ctrl + LV-GFP + Vehicle = 1.155 ± 0.2648 Ctrl + LV-siCD200R1 + Vehicle = 1.820 ± 0.2061 CSDS + LV-GFP + Vehicle = 0.5421 ± 0.1059 CSDS + LV-siCD200R1 + Vehicle = 0.4765 ± 0.08037 Ctrl + LV-GFP + CD200Fc = 1.067 ± 0.1222 Ctrl + LV-siCD200R1 + CD200Fc = 1.520 ± 0.1586 CSDS + LV-GFP + CD200Fc = 1.040 ± 0.1838 CSDS + LV-siCD200R1 + CD200Fc = 0.5402 ± 0.1002 |
| 4G Ctrl + LV-GFP + Vehicle = 6 Ctrl + LV-siCD200R1 + Vehicle = 8 CSDS + LV-GFP + Vehicle = 10 CSDS + LV-siCD200R1 + Vehicle = 11 Ctr l+ LV-GFP + CD200Fc = 11 Ctrl + LV-siCD200R1 + CD200Fc = 8 CSDS + LV-GFP + CD200Fc = 8 CSDS + LV-siCD200R1 + CD200Fc = 11 | One-way ANOVA Treatment Factor: F (7, 65) = 15.97/P<0.001 | Fisher's LSD post-test Ctrl + LV-GFP + Vehicle vs. CSDS + LV-GFP + Vehicle P<0.001 Ctrl + LV-siCD200R1 + Vehicle vs. CSDS + LV-siCD200R1 + Vehicle P<0.001 Ctrl + LV-GFP + CD200Fc vs. CSDS + LV-GFP + CD200Fc P=0.112 Ctrl + LV-siCD200R1 + CD200Fc vs. CSDS + LV-siCD200R1 + CD200Fc P=0.007 | Ctrl + LV-GFP + Vehicle = 115.8 ± 9.913 Ctrl + LV-siCD200R1 + Vehicle = 117.1 ± 13.52 CSDS + LV-GFP + Vehicle = 231.9 ± 8.084 CSDS + LV-siCD200R1 + Vehicle = 219.8 ± 14.76 Ctrl + LV-GFP + CD200Fc = 127.8 ± 15.78 Ctrl + LV-siCD200R1 + CD200Fc = 104.4 ± 16.71 CSDS + LV-GFP + CD200Fc = 97.75 ± 11.54 CSDS + LV-siCD200R1 + CD200Fc = 156.5 ± 10.14 |
| 4H Ctrl + LV-GFP + Vehicle = 6 Ctrl + LV-siCD200R1 + Vehicle = 8 CSDS + LV-GFP + Vehicle = 9 CSDS+LV-siCD200R1+Vehicle = 11 Ctrl+LV-GFP+CD200Fc = 11 Ctrl+LV-siCD200R1+CD200Fc = 7 CSDS+LV-GFP+CD200Fc = 7 CSDS+LV-siCD200R1+CD200Fc = 10 | One-way ANOVA Treatment Factor: F (7, 61) = 9.369/P<0.001 | Fisher's LSD post-test Ctrl + LV-GFP + Vehicle vs. CSDS + LV-GFP + Vehicle P<0.001 Ctrl + LV-siCD200R1 + Vehicle vs. CSDS+LV-siCD200R1 + Vehicle P<0.001 Ctrl + LV-GFP + CD200Fc vs. CSDS + LV-GFP + CD200Fc P=0.067 Ctrl + LV-siCD200R1 + CD200Fc vs. CSDS + LV-siCD200R1 + CD200Fc P=0.037 | Ctrl + LV-GFP + Vehicle = 68.68 ± 15.79 Ctrl + LV-siCD200R1 + Vehicle = 56.29 ± 9.721 CSDS + LV-GFP + Vehicle = 153.6 ± 15.72 CSDS + LV-siCD200R1 + Vehicle = 169.2 ± 13.16 Ctrl + LV-GFP + CD200Fc = 106.5 ± 11.91 Ctrl + LV-siCD200R1 + CD200Fc = 70.71 ± 12.17 CSDS + LV-GFP + CD200Fc = 69.43 ± 15.58 CSDS + LV-siCD200R1 + CD200Fc = 113.9 ± 15.54 |
| 4I Ctrl + LV-GFP + Vehicle = 6 Ctrl + LV-siCD200R1 + Vehicle = 8 CSDS + LV-GFP + Vehicle = 10 CSDS + LV-siCD200R1 + Vehicle = 11 Ctrl + LV-GFP + CD200Fc = 11 Ctrl + LV-siCD200R1 + CD200Fc = 8 CSDS+LV-GFP+CD200Fc = 8 CSDS + LV-siCD200R1 + CD200Fc = 11 | One-way ANOVA Treatment Factor: F (7, 65) = 2.061/P=0.061 | Fisher's LSD post-test Ctrl + LV-GFP + Vehicle vs. CSDS + LV-GFP + Vehicle P=0.066 Ctrl + LV-siCD200R1 + Vehicle vs. CSDS+LV-siCD200R1 + Vehicle P=0.246 Ctrl + LV-GFP + CD200Fc vs. CSDS + LV-GFP + CD200Fc P=0.132 Ctrl + LV-siCD200R1 + CD200Fc vs. CSDS+LV-siCD200R1 + CD200Fc P=0.494 | Ctrl + LV-GFP + Vehicle =4551 ± 311.1 Ctrl + LV-siCD200R1 + Vehicle = 4232 ± 405.5 CSDS + LV-GFP + Vehicle = 3242 ± 264.1 CSDS + LV-siCD200R1 + Vehicle = 3494 ± 327.5 Ctrl + LV-GFP + CD200Fc = 3876 ± 405.7 Ctrl + LV-siCD200R1 + CD200Fc = 5011 ± 535.5 CSDS + LV-GFP + CD200Fc = 4838 ± 734.0 CSDS + LV-siCD200R1 + CD200Fc = 4578 ± 477.6 |
| 5C Ctrl + Vehicle = 3 Ctrl + CD200Fc = 3 CSDS + Vehicle = 3  CSDS + CD200Fc = 3 | Two-way ANOVA Interaction Factor: F (1, 32) = 8.187/P=0.007 CD200Fc Factor: F (1, 32) = 3.311/P=0.078 CSDS Factor: F (1, 32) = 2.355/P=0.135 | Fisher's LSD post-test Ctrl: Vehicle vs. CSDS: Vehicle/ P=0.002 CSDS: Vehicle vs. CSDS: CD200Fc/ P=0.004 | Ctrl + Vehicle = 119.3 ± 10.06 Ctrl + CD200Fc = 143.4 ± 18.24 CSDS + Vehicle = 204.4 ± 28.35  CSDS + CD200Fc = 124.4 ± 9.332 |
| 5D Ctrl + Vehicle = 3 Ctrl + CD200Fc = 3 CSDS + Vehicle = 3  CSDS + CD200Fc = 3 | Two-way ANOVA Interaction Factor: F (1, 32) = 0.1482/P=0.703 CD200Fc Factor: F (1, 32) = 10.21/P=0.003 CSDS Factor: F (1, 32) = 15.69/P<0.001 | Fisher's LSD post-test Ctrl: Vehicle vs. CSDS: Vehicle/ P=0.016 CSDS: Vehicle vs. CSDS: CD200Fc/ P=0.004 | Ctrl + Vehicle = 279.3 ± 23.58 Ctrl + CD200Fc = 366.6 ± 31.10 CSDS + Vehicle = 192.0 ± 21.67  CSDS + CD200Fc = 298.0 ± 19.65 |
| 5E Ctrl + Vehicle = 3 Ctrl + CD200Fc = 3 CSDS + Vehicle = 3  CSDS + CD200Fc = 3 | Two-way ANOVA Interaction Factor: F (24, 288) = 2.548/P<0.001 CD200Fc Factor: F (8, 288) = 42.72/P<0.001 CSDS Factor: F (3, 288) = 13.61/P<0.001 | Fisher's LSD post-test  12μm: CSDS + Vehicle vs. CSDS+CD200Fc/ P=0.003 15μm: CSDS + Vehicle vs. CSDS+CD200Fc/ P=0.004 18μm: CSDS + Vehicle vs. CSDS+CD200Fc/ P=0.016 | 12μm: CSDS + Vehicle: 7.222 ± 0.8941 15μm: CSDS + Vehicle: 5.111 ± 0.7536 18μm: CSDS + Vehicle: 3.778 ± 0.7222 12μm: CSDS + CD200Fc: 11.33 ± 1.130 15μm: CSDS + CD200Fc: 9.111 ± 0.9346 18μm: CSDS + CD200Fc: 7.111 ± 0.9196 |
| 5G Ctrl+ Vehicle = 3 Ctrl + CD200Fc = 3 CSDS + Vehicle = 3  CSDS + CD200Fc = 3 | Two-way ANOVA Interaction Factor: F (1, 8) = 16.19/P=0.004 CD200Fc Factor: F (1, 8) = 29.09/P<0.001 CSDS Factor: F (1, 8) = 25.25/P=0.001 | Fisher's LSD post-test Ctrl: Vehicle vs. CSDS: Vehicle/ P<0.001 CSDS: Vehicle vs. CSDS: CD200Fc/ P<0.001 | Ctrl + Vehicle = 102.9 ± 6.727 Ctrl + CD200Fc = 90.00 ± 0.5774 CSDS + Vehicle = 224.3 ± 21.23 CSDS + CD200Fc = 107.7 ± 12.99 |
| 5H Ctrl + Vehicle = 3 Ctrl + CD200Fc = 3 CSDS + Vehicle = 3  CSDS + CD200Fc = 3 | Two-way ANOVA Interaction Factor: F (1, 22) = 8.119/P=0.009 CD200Fc Factor: F (1, 22) = 7.837/P=0.010 CSDS Factor: F (1, 22) = 12.18/P=0.002 | Fisher's LSD post-test Ctrl: Vehicle vs. CSDS: Vehicle/ P<0.001 CSDS: Vehicle vs. CSDS: CD200Fc/ P<0.001 | Ctrl + Vehicle = 0.7041 ± 0.1427 Ctrl + CD200Fc = 0.5128 ± 0.1000 CSDS + Vehicle = 2.391 ± 0.6074  CSDS + CD200Fc = 0.4978 ± 0.04233 |
| 5J Ctrl + Vehicle = 3 Ctrl + CD200Fc = 3 CSDS + Vehicle = 3  CSDS + CD200Fc = 3 | Two-way ANOVA Interaction Factor: F (1, 44) = 2.185/P=0.146 CD200Fc Factor: F (1, 44) = 3.014/P=0.090 CSDS Factor: F (1, 44) = 31.39/P<0.001 | Fisher's LSD post-test Ctrl: Vehicle vs. CSDS: Vehicle/ P=0.028 CSDS: Vehicle vs. CSDS: CD200Fc/ P<0.001 | Ctrl + Vehicle = 1.242 ± 0.1975 Ctrl + CD200Fc = 1.905 ± 0.1452 CSDS + Vehicle = 0.7260 ± 0.1533  CSDS + CD200Fc = 1.863 ± 0.1400 |
| 6D Ctrl + AAV-GFP = 3 Ctrl + AAV-CD200 = 3 CSDS + AAV-GFP = 3  CSDS + AAV-CD200 = 3 | Two-way ANOVA Interaction Factor: F (1, 8) = 1.855/P=0.210 AAV-CD200 Factor: F (1, 8) = 4.216/P=0.074 CSDS Factor: F (1, 8) = 7.882/P=0.023 | Fisher's LSD post-test Ctrl: AAV-GFP vs. CSDS: AAV-GFP/ P=0.042 CSDS: AAV-GFP vs. CSDS: AAV-CD200/ P=0.018 | Ctrl + AAV-GFP = 62.00 ± 4.880 Ctrl + AAV-CD200 = 69.67 ± 9.264 CSDS + AAV-GFP = 43.89 ± 1.544 CSDS + AAV-CD200 =66.00 ± 0.6939 |
| 6E Ctrl + AAV-GFP = 3 Ctrl + AAV-CD200 = 3 CSDS + AAV-GFP = 3  CSDS + AAV-CD200 = 3 | Two-way ANOVA Interaction Factor: F (1, 8) = 0.1381/P=0.720 AAV-CD200 Factor: F (1, 8) = 13.42/P=0.006 CSDS Factor: F (1, 8) = 24.03/P=0.001 | Fisher's LSD post-test Ctrl: AAV-GFP vs. CSDS: AAV-GFP/ P=0.021 CSDS: AAV-GFP vs. CSDS: AAV-CD200/ P=0.006 | Ctrl + AAV-GFP = 24.11 ± 0.9686 Ctrl + AAV-CD200 = 31.22 ± 2.231 CSDS + AAV-GFP = 17.78 ± 0.4006 CSDS+AAV-CD200 = 26.06 ± 1.944 |
| 6G Ctrl + AAV-GFP = 3 Ctrl + AAV-CD200 = 3 CSDS + AAV-GFP = 3  CSDS + AAV-CD200 = 3 | Two-way ANOVA Interaction Factor: F (1, 8) = 20.84/P=0.002 AAV-CD200 Factor: F (1, 8) = 18.68/P=0.003 CSDS Factor: F (1, 8) = 19.03/P=0.002 | Fisher's LSD post-test Ctrl: AAV-GFP vs. CSDS: AAV-GFP/ P<0.001 CSDS: AAV-GFP vs. CSDS: AAV-CD200/ P<0.001 | Ctrl + AAV-GFP = 26.02 ± 0.8453 Ctrl + AAV-CD200 = 25.82 ± 1.355 CSDS + AAV-GFP = 17.42 ± 0.1502 CSDS + AAV-CD200 = 26.06 ± 1.107 |
| 6H Ctrl + AAV-GFP = 3 Ctrl + AAV-CD200 = 3 CSDS + AAV-GFP = 3  CSDS + AAV-CD200 = 3 | Two-way ANOVA Interaction Factor: F (1, 8) = 4.671/P=0.063 AAV-CD200 Factor: F (1, 8) = 1.442/P=0.264 CSDS Factor: F (1, 8) = 13.58/P=0.006 | Fisher's LSD post-test Ctrl: AAV-GFP vs. CSDS: AAV-GFP/ P=0.045 CSDS: AAV-GFP vs. CSDS: AAV-CD200/ P=0.003 | Ctrl + AAV-GFP = 6.778 ± 0.4006 Ctrl + AAV-CD200 = 7.792 ± 0.7372 CSDS + AAV-GFP = 4.542 ± 0.2083 CSDS + AAV-CD200 = 8.431 ± 1.011 |
| 6J Ctrl + AAV-GFP = 4 Ctrl + AAV-CD200 = 5 CSDS + AAV-GFP = 3  CSDS + AAV-CD200 = 3 | Two-way ANOVA Interaction Factor: F (1, 11) = 4.967/P=0.054 AAV-CD200 Factor: F (1, 11) = 22.60/P<0.001 CSDS Factor: F (1, 11) = 44.50/P<0.001 | Fisher's LSD post-test Ctrl: AAV-GFP vs. CSDS: AAV-GFP/ P<0.001 CSDS: AAV-GFP vs. CSDS: AAV-CD200/ P<0.001 | Ctrl + AAV-GFP = 18.22 ± 0.2948 Ctrl + AAV-CD200 = 22.90 ± 1.358 CSDS + AAV-GFP = 11.04 ± 0.8365 CSDS+AAV-CD200 = 20.21 ± 0.2320 |
| 6K Ctrl + AAV-GFP = 4 Ctrl + AAV-CD200 = 5 CSDS + AAV-GFP = 3  CSDS + AAV-CD200 = 3 | Two-way ANOVA Interaction Factor: F (1, 11) = 4.295/P=0.063 AAV-CD200 Factor: F (1, 11) = 23.47/ P<0.001 CSDS Factor: F (1, 11) = 30.51/ P<0.001 | Fisher's LSD post-test Ctrl: AAV-GFP vs. CSDS: AAV-GFP/ P<0.001 CSDS: AAV-GFP vs. CSDS: AAV-CD200/ P<0.001 | Ctrl + AAV-GFP = 10.78 ± 0.3361 Ctrl + AAV-CD200 = 13.38 ± 0.6374 CSDS + AAV-GFP = 5.583 ± 0.7784 CSDS + AAV-CD200 = 11.29 ± 1.234 |
| S1A Control = 9 CSDS = 9 | Control vs. CSDS /t-test/ p=0.280 |  | Control = 1.000 ± 0.06482 CSDS = 0.9113 ± 0.04579 |
| S1B Control = 6 CSDS = 10 | Control vs. CSDS /t-test/ p=0.021 |  | Control = 1.000 ± 0.1094 CSDS = 0.7636 ± 0.02950 |
| S1C Control = 7 CSDS = 9 | Control vs. CSDS /t-test/ P<0.001 |  | Control = 1.023 ± 0.02825 CSDS = 0.7161 ± 0.04285 |
| S1D Control = 6 CSDS = 9 | Control vs. CSDS /t-test/ P=0.091 |  | Control = 1.027 ± 0.05295 CSDS = 0.9021 ± 0.04315 |
| S1E Control = 5 CSDS = 6 | Control vs. CSDS /t-test/ P=0.828 |  | Control = 1.000 ± 0.02839 CSDS = 1.023 ± 0.08879 |
| S2B Ctrl + Vehicle = 9  Ctrl + CD200Fc = 9 CSDS + Vehicle = 9  CSDS+CD200Fc = 9 | Two-way ANOVA Interaction Factor: F (1, 32) = 2.787/P=0.105 CD200 Factor: F (1, 32) = 12.66/P=0.001 Stress Factor: F (1, 32) = 4.633/P=0.039 | Fisher's LSD post-test CSDS: Vehicle vs. Ctrl: Vehicle/ P<0.001 CSDS:CD200Fc vs. CSDS: Vehicle/ P=0.011 | Ctrl + Vehicle = 1.083 ± 0.07640 Ctrl + CD200Fc = 1.133 ± 0.07205 CSDS + Vehicle = 0.5494 ± 0.1397 CSDS + CD200Fc = 0.9397 ± 0.1056 |
| S2C Ctrl + Vehicle = 10  Ctrl + CD200Fc = 8 CSDS + Vehicle = 11  CSDS + CD200Fc = 10 | Two-way ANOVA Interaction Factor: F (1, 35) = 9.247/P=0.004 CD200 Factor: F (1, 35) = 0.07364/P=0.788 Stress Factor: F (1, 35) = 8.590/P=0.006 | Fisher's LSD post-test CSDS: Vehicle vs. Ctrl: Vehicle/ P=0.049 CSDS: CD200Fc vs. CSDS: Vehicle/ P<0.001 | Ctrl + Vehicle = 122.6 ± 11.09 Ctrl + CD200Fc = 124.1 ± 13.07 CSDS + Vehicle = 161.0 ± 10.67 CSDS+CD200Fc = 78.20 ± 18.72 |
| S2D Ctrl + Vehicle = 10  Ctrl + CD200Fc = 9 CSDS + Vehicle = 12  CSDS + CD200Fc = 12 | Two-way ANOVA Interaction Factor: F (1, 39) = 1.046/P=0.313 CD200 Factor: F (1, 39) = 3.605/P=0.065 Stress Factor: F (1, 39) = 13.45/P<0.001 | Fisher's LSD post-test CSDS: Vehicle vs. Ctrl: Vehicle/ P=0.043 CSDS:CD200Fc vs. CSDS: Vehicle/ P=0.001 | Ctrl + Vehicle = 106.7 ± 9.350 Ctrl + CD200Fc = 77.00 ± 13.01 CSDS + Vehicle = 139.5 ± 8.750 CSDS + CD200Fc = 86.83 ± 12.88 |
| S2E Ctrl + Vehicle = 12  Ctrl+CD200Fc = 9 CSDS + Vehicle = 12  CSDS+CD200Fc = 12 | Two-way ANOVA Interaction Factor: F (1, 41) = 0.1198/P=0.731 CD200 Factor: F (1, 41) = 1.635/P=0.208 Stress Factor: F (1, 41) = 3.534/P=0.067 | Fisher's LSD post-test CSDS: Vehicle vs. Ctrl: Vehicle/ P=0.239 CSDS:CD200Fc vs. CSDS: Vehicle/ P=0.109 | Ctrl +Vehicle = 3616 ± 335.7 Ctrl+CD200Fc = 4204 ± 579.1 CSDS + Vehicle = 2993 ± 252.2 CSDS +CD200Fc = 3847 ± 382.8 |
| S3B Arg-1 CSDS + AAV-GFP = 6 CSDS + AAV-CD200 = 6 | CSDS+AAV-GFP vs. CSDS+AAV-CD200 /t-test/ p=0.0494 |  | CSDS+AAV-GFP = 1.000 ± 0.1563 CSDS+AAV-CD200 = 1.588 ± 0.2113 |
| S3B YM-1 CSDS+AAV-GFP = 6 CSDS+AAV-CD200 = 5 | CSDS+AAV-GFP vs. CSDS+AAV-CD200 /t-test/ p=0.009 |  | CSDS+AAV-GFP = 1.000 ± 0.1918 CSDS+AAV-CD200 = 2.033 ± 0.2488 |
| S3B IL-4 CSDS+AAV-GFP = 6 CSDS+AAV-CD200 = 6 | CSDS+AAV-GFP vs. CSDS+AAV-CD200 /t-test/ p=0.033 |  | CSDS+AAV-GFP = 1.000 ± 0.1425 CSDS+AAV-CD200 = 2.303 ± 0.5096 |
| S3B TNF-α CSDS + AAV-GFP = 5 CSDS + AAV-CD200 = 5 | CSDS+AAV-GFP vs. CSDS+AAV-CD200 /t-test/ p=0.030 |  | CSDS+AAV-GFP = 1.000 ± 0.1080 CSDS+AAV-CD200 = 0.6379 ± 0.08443 |
| S3B IL-6 CSDS + AAV-GFP = 6 CSDS + AAV-CD200 = 6 | CSDS+AAV-GFP vs. CSDS+AAV-CD200 /t-test/ p=0.244 |  | CSDS+AAV-GFP = 1.000 ± 0.1890 CSDS+AAV-CD200 = 1.304 ± 0.1569 |
| S3B IL-1β CSDS + AAV-GFP = 6 CSDS + AAV-CD200 = 6 | CSDS+AAV-GFP vs. CSDS+AAV-CD200 /t-test/ p=0.691 |  | CSDS+AAV-GFP = 1.000 ± 0.2321 CSDS+AAV-CD200 = 1.116 ± 1.535 |
| S4C Ctrl + Vehicle = 3  Ctrl + CD200Fc = 3 CSDS + Vehicle = 3  CSDS + CD200Fc = 3 | Two-way ANOVA Interaction Factor: F (1, 32) = 3.644/P=0.065 CD200 Factor: F (1, 32) = 3.371/P=0.076 Stress Factor: F (1, 32) = 16.59/P<0.001 | Fisher's LSD post-test CSDS: Vehicle vs. Ctrl: Vehicle/ P=0.012 CSDS: CD200Fc vs. CSDS: Vehicle/ P<0.001 | Ctrl + Vehicle = 2.786 ± 0.4605 Ctrl + CD200Fc = 3.782 ± 0.6276 CSDS + Vehicle = 1.063 ± 0.1926 CSDS+CD200Fc = 3.815 ± 0.4512 |
| S5C Ctrl + Vehicle = 4  Ctrl + CD200Fc = 3 CSDS + Vehicle = 5  CSDS + CD200Fc = 3 | Two-way ANOVA Interaction Factor: F (1, 11) = 1.965/P=0.189 CD200 Factor: F (1, 11) = 7.555/P=0.019 Stress Factor: F (1, 11) = 137.7/P<0.001 | Fisher's LSD post-test CSDS: Vehicle vs. Ctrl: Vehicle/ P=0.007 CSDS:CD200Fc vs. CSDS: Vehicle/ P<0.001 | Ctrl + Vehicle = 11.09 ± 1.017 Ctrl + CD200Fc = 24.63 ± 0.1909 CSDS + Vehicle = 5.658 ± 0.5398 CSDS + CD200Fc = 22.86 ± 2.861 |
| S5D Ctrl + Vehicle = 4  Ctrl+CD200Fc = 3 CSDS + Vehicle = 5  CSDS + CD200Fc = 3 | Two-way ANOVA Interaction Factor: F (1, 11) = 0.7358/P=0.409 CD200 Factor: F (1, 11) = 3.773/P=0.078 Stress Factor: F (1, 11) = 47.83/P<0.001 | Fisher's LSD post-test CSDS: Vehicle vs. Ctrl: Vehicle/ P=0.050 CSDS: CD200Fc vs. CSDS: Vehicle/ P<0.001 | Ctrl + Vehicle = 5.219 ± 0.8171 Ctrl + CD200Fc = 11.35 ± 1.210 CSDS + Vehicle = 2.400 ± 0.3903 CSDS + CD200Fc = 10.25 ± 1.843 |
